# The Potential of Plant Secondary Metabolites as Bread Mould Inhibitors: Exploring Their Individual and Combined Antifungal Effect

**DOI:** 10.3390/foods14213604

**Published:** 2025-10-23

**Authors:** Amber Lepoutre, Els Debonne, Wouter Van Genechten, Serena Martini, Patrick Van Dijck, Frank Devlieghere

**Affiliations:** 1 Research Unit Food Microbiology and Food Preservation, Department of Food Technology, Safety and Health, Faculty of Bioscience Engineering, Ghent University, Coupure Links 653, 9000 Ghent, Belgium; amber.lepoutre@ugent.be (A.L.);; 2 Laboratory of Molecular Cell Biology, Department of Biology, Institute of Botany and Microbiology, KU Leuven, Kasteelpark Arenberg 31, 3001 Leuven, Belgiumpatrick.vandijck@kuleuven.be (P.V.D.); 3 Kemin Food Technologies, EMEA, Via Francesco Pescatori, 4i, 43126 Parma, Italy

**Keywords:** plant secondary metabolites, benzyl isothiocyanate, carvacrol, *Penicillium* spp., *Aspergillus* spp., bread spoilage, mould inhibitor

## Abstract

Plant secondary metabolites are an interesting source of natural antifungals and offer an alternative to synthetic preservatives. In this study, the activity of 218 secondary metabolites was evaluated against nine *Penicillium* species and one *Aspergillus* species, isolated from spoiled par-baked bread. By comparing agar and liquid-based assays, it was found that the hydrophobic nature of these compounds led to an underestimation of the activity in agar-based assays. In liquid medium, it was possible to evaluate the effect quantitatively and differentiate between strong and weak inhibitors. Of the most interesting compounds, the minimal inhibitory concentration (MIC) was determined, and synergistic interactions were studied. This revealed an interesting interaction between benzyl isothiocyanate and carvacrol, which was further investigated through validation in par-baked bread. Antifungal efficacy was assessed in a shelf life and challenge test, revealing that spray application of 200 to 400 µg/mL benzyl isothiocyanate and 1000 to 2000 µg/mL carvacrol significantly increased shelf life. Furthermore, application of benzyl isothiocyanate and carvacrol was as effective as 0.15% propionic acid was incorporated in the dough. A sensory triangle test indicated that benzyl isothiocyanate and carvacrol influenced the flavour of fully baked bread; however, the effect was not perceived negatively.

## 1. Introduction

Par-baked bread is a convenient technology that interrupts the baking process after crumb formation but before forming a crispy and coloured crust. This minimizes the staling process, resulting in an increased shelf life and providing consumers with freshly baked products [1]. However, these products are still prone to microbiological spoilage, thereby having an economic impact and affecting food safety [2].

Spoilage of par-baked bread is dominated by *Penicillium* and *Aspergillus* species [3,4,5,6,7,8] and to a lesser extent by *Alternaria* species, *Eurotium* species and chalk moulds [4,5,9,10]. By implementing modified atmosphere packaging and addition of synthetic preservatives, the shelf life of par-baked bread is prolonged from several days to several weeks [11]. However, a trend in consumer’s behaviour towards products free from synthetic preservatives has been observed [12]. To ensure food safety and reduce food waste, replacement of synthetic preservatives by natural antifungal compounds is explored.

An interesting source of natural preservatives are plant secondary metabolites, especially essential oils. These compounds are involved in host defence mechanisms against pathogen infections [13,14]. Essential oils are a mixture of many essential oil compounds that can be divided in four different groups of compounds: terpenes, terpenoids, phenylpropenes and others [13]. The composition of essential oils varies according to the growth conditions, the part of the plant and the extraction method, making it difficult to compare research findings [14]. The lipophilic character of essential oil (compounds) facilitates passage through the cell membrane and enables membrane permeabilization and inhibition of intracellular targets [15].

Growth inhibition by essential oils results from the combined activity of multiple compounds acting on the microbial cell, often leading to membrane disruption. Their mode of action cannot be attributed to one single mechanism but rather to a combination of effects on multiple targets [16]. The specific mode of action depends on the functional groups present in each compound. For example, compounds such as carvacrol and thymol possess an aliphatic chain that enables interaction with the cell membrane, while the phenolic hydroxyl group functions as a proton exchanger [17]. This interaction increases membrane permeability, disrupts the proton motive force and impairs ATP synthesis [17]. Compounds lacking the methyl, isopropyl or hydroxyl group have been reported to be less active than carvacrol, likely due to a reduced amphipathic character or decreased acidity [18]. Compounds with an isothiocyanate group, such allyl and benzyl isothiocyanate, can interact with thiol groups. Isothiocyanates have been found to alter protein structure and function by interacting with the thiol group of cysteine, thereby affecting the metabolism, electron transport and stress response of the microbial cell [19]. Combining compounds with different mode of actions may result in a synergistic antimicrobial effect. Synergy can result from the complementary actions of two compounds on multiple targets within a biochemical pathway, their simultaneous action on the cell membrane, or the interaction of one compound enhancing the uptake of the other compound by increasing membrane permeability or decreasing drug efflux [20,21].

Depending on the method of application within the food matrix, it can be useful to assess the in vitro antimicrobial activity using diffusion or dilution assays. In vitro dilution assays provide insight into the antimicrobial activity of a compound when it is dispersed within the medium, which is relevant for its incorporation into the dough [13]. When antimicrobials are incorporated into an active packaging system, their efficacy depends on the activity in the vapour phase and can therefore be evaluated through in vitro diffusion assays. Spray application, on the other hand, relies on both diffusion and dilution behaviour, and information obtained from both types of assays can be relevant [13].

Despite considerable research efforts to develop a natural preservation strategy for bread products, these efforts have not yet resulted in a commercially applied strategy. On the one hand, this can be attributed to the insufficient validation of promising in vitro results in a real food matrix. On the other hand, studies on the validation in real food matrices report a reduced antimicrobial efficacy in complex food systems, requiring higher concentrations than those effective during in vitro experiments. Consequently, these higher concentrations negatively affect the physicochemical and sensorial properties of the final product [22,23,24]. A possible solution to eliminate or minimize this impact is by combining compounds with a synergistic effect. Synergism would allow the addition of a lower dosage of the compound while maintaining its antimicrobial activity [20].

In addition, the method of application can influence the impact on the product’s properties. Most studies report the validation of antimicrobial compounds through incorporation into the dough. This is a straightforward method that does not require additional equipment. However, incorporating antimicrobials into the dough can affect the leavening capacity of the baker’s yeast *Saccharomyces cerevisiae*, resulting in a reduced bread volume [22]. Furthermore, volatile or heat-sensitive compounds may evaporate or degrade during the baking process. Alternatively, antimicrobial compounds can be applied on the bread’s surface through spray application or incorporated into an active packaging system. In spray application, antimicrobials are applied directly on the bread’s surface instead of being dispersed throughout the bread matrix. Consequently, less material is required compared to dough incorporation, and the compounds are directly applied on the area of concern. Application of essential oils via spray application has been reported to be more effective than incorporation into the dough [25]. When incorporated into an active packaging system, the antimicrobial effect depends on the diffusion of volatile compounds from the packaging material to the food product, unless the system is in direct contact with the product [26]. The efficacy of an active packaging system greatly depends on the specific system and production process. Ideally, the system should allow a high loading capacity, good storage stability and a controlled, gradual release of the active compound. The processing method should be non-thermal to prevent the evaporation and degradation of volatile and heat-sensitive compounds and should preferably avoid the use of organic solvents [26].

In this research, the goal was to identify plant secondary metabolites active against bread moulds isolated from spoiled par-baked bread. By using pure plant secondary metabolites, rather than essential oils or plant extracts, it was possible to identify the compounds responsible for antimicrobial activity. All plant secondary metabolites included in this study are allowed to be used in food according to the European Food Safety Authority (EFSA). Nine different *Penicillium* and one *Aspergillus* species were included to ensure broad-spectrum activity, and antifungal activity was evaluated in both agar-based and liquid-based methods. The antifungal effect of the most interesting compounds was further explored to unravel the minimal inhibitory concentration and possible synergistic effects. The most interesting combination, benzyl isothiocyanate combined with carvacrol, was further validated in a par-baked bread matrix. The antifungal efficacy was assessed through spray application of the compounds on the surface of par-baked bread. In addition, the impact of benzyl isothiocyanate and carvacrol on the colour parameters of the surface of the bread, as well as the sensorial quality, was evaluated.

## 2. Materials and Methods

### 2.1. Fungal Isolates

The moulds used in this research were isolated from spoiled par-baked bread, which were packaged under modified atmosphere and produced in Western Europe [8]. *Penicillium polonicum* (AL_01), *Penicillium crustosum* (AL_03), *Penicillium brevicompactum* (AL_04), *Aspergillus westerdijkiae* (AL_10), *Penicillium chrysogenum* (AL_17), *Penicillium palitans* (AL_37), *Penicillium bialowiezense* (AL_57), *Penicillium glabrum* (AL_67), *Penicillium corylophilum* (AL_72) and *Penicillium hordei* (AL_75) are part of the culture collection of the Laboratory of Molecular Cell Biology, Department of Biology (KU Leuven, Leuven, Belgium). All strains are stored at −80 °C.

### 2.2. Plant Secondary Metabolites

Supplementary Table S1 lists the plant secondary metabolites used in this research and their supplier. Plant secondary metabolites were selected based on their approval for use in food products, according to the European Food Safety Authority (EFSA). In addition, the selection aimed to cover a wide range of chemical classes by including both compounds with reported antimicrobial activity, which are often major constituents of plant extracts, as well as compounds occurring in lower concentrations within the extracts. The importance of including both major and minor constituents of plant extracts was motivated by earlier findings reported by Feyaerts et al. [27]. All compounds were stored in the dark, at either room temperature, 4 °C or −20 °C, according to the information provided by the supplier.

### 2.3. Media Preparation

To maintain the moulds and to perform the agar disc diffusion assay, potato dextrose agar (PDA) plates were used. The PDA plates contained 4 g/L potato extract (Formedium, Norfolk, England), 15 g/L Difco agar (BD, Biosciences, NJ, U.S.) and 20 g/L glucose (Sigma-Aldrich, St. Louis, MO, U.S.). The medium was autoclaved for fifteen minutes at 121 °C and poured into Petri dish plates. In the agar disc diffusion assay, each plate contained 20 mL of PDA.

Semi-solid yeast extract sucrose (YES) medium was used in an antifungal susceptibility test in liquid medium, the minimal inhibitory concentration (MIC) assay and the checkerboard assay. The preparation of the medium was based on Debonne et al. [22] and contained 20 g/L yeast extract (Merck, Darmstadt, Germany), 150 g/L sucrose (Sigma-Aldrich), 1 g/L magnesium sulphate (Sigma-Aldrich) and 1.2 g/L Difco agar (BD, Biosciences). The medium was prepared per 300 mL and autoclaved for fifteen minutes at 121 °C.

### 2.4. Inoculum Preparation

The method used for the inoculum preparation was based on Debonne et al. [23]. All strains were plated and maintained on PDA plates during the experiments. One week before each experiment, the fungal spores were transferred to a fresh PDA plate and incubated at 26 °C. After seven days, 5 mL of cold sterile deionized water with 0.1% Tween 80 (Sigma-Aldrich) was added to the plate and the fungal material was scraped loose. Then, the solution was transferred to a sterile cotton filter and collected in a sterile falcon tube. This step was repeated three times. Then, the cotton filter was discarded, and the filtrate solution was centrifuged (Allegra X-15R) for fifteen minutes at 3273 g and 4 °C. After removing the supernatant, the pellet was resuspended in 25 mL cold sterile PBS (10×) (80 g/L NaCl (Sigma-Aldrich), 2 g/L (VWR, Radnor, PA, U.S.), 14.4 g/L Na_2_HPO_4_ (Merck), 2.4 g/L KH_2_PO_4_ (Merck)) with 0.1% Tween 80. Again, the solution was centrifuged for fifteen minutes at 3273× *g* and 4 °C and the supernatant was removed. Then, the pellet was resuspended in PBS (1×) and the spore concentration was determined microscopically using a Bürker chamber.

### 2.5. Agar Disc Diffusion Assay

The agar disc diffusion method was performed according to the protocol published by the Clinical and Laboratory Standards Institute (M44-A) [28], with some slight modifications. All plant secondary metabolites listed in Supplementary Table S1 were tested. Fresh PDA plates with a diameter of 90 mm and an agar depth of 4 mm were inoculated with 10^5^ spores. A Whatman filter paper disc with 6 mm diameter was placed at the centre of the inoculated PDA plates, and 5 µL of a plant secondary metabolite (1% solution, diluted in ethanol) was administered onto the paper disc. Dilutions were prepared in microcentrifuge tubes, which were vortexed thoroughly before applying the compound to the filter paper. After application, the plates were closed immediately and incubated for 48 h at 26 °C. After incubation, images of the plates were made using a flatbed scanner, and the diameter of the inhibition zones was measured using ImageJ version 1.53k [29].

### 2.6. Antifungal Susceptibility Testing

All plant secondary metabolites listed in Supplementary Table S1 were tested for antifungal activity in liquid broth against the ten bread moulds included in this research. This screening was performed according to the protocol published by the European Committee on Antimicrobial Susceptibility Testing [30], with some modifications. In this experiment, the compounds were tested at one fixed concentration. Both the compounds and the spores were diluted in semi-solid YES medium and added to 96-well plates to obtain a final concentration of 500 µg/mL and 1000 spores/well, respectively, with a total volume of 200 µL in each well. Every compound was tested in duplicate and a control containing only inoculated semi-solid YES medium was taken along, as well as a control with ethanol (2.5%). The outer wells of the 96-well plates were filled with 200 µL semi-solid YES medium to minimize evaporation of the inner wells during incubation. The plates were sealed using a Breathe-Easy™ film (Sigma-Aldrich) and incubated for 48 h at 26 °C. Due to the volatile and hydrophobic character, dilutions were prepared in microcentrifuge tubes and vortexed thoroughly before being added to 96-well plates. No more than three minutes passed between the addition of the first and the last compound, and the plate was immediately sealed afterwards. The same procedure was applied for the MIC and checkerboard assays. Every 24 h, the optical density (OD) was measured at 595 nm. During the first 24 h, the increase in OD was due to evaporation of the medium on the seal, as a similar increase was also measured in wells containing blank semi-solid YES medium. After 24 h, no further increase in OD due to evaporation was observed. The absence of growth in the first 24 h was confirmed by growth curves (Supplementary Figure S1). Antifungal activity was expressed as the mean percentage of growth inhibition of the two technical repeats, relative to the control containing only inoculated semi-solid YES medium. The effect of the medium and evaporation on the OD was taken into account by subtracting the OD_595_ measured at 24 h (Equation (1)). For each compound, two technical replicates were included against each mould strain. It was observed that, at the tested concentration, ethanol did not affect the growth of the moulds. Therefore, this control was not taken along in the minimal inhibitory concentration assay and checkerboard assay.

(1) $$Growth\;inhibition=100-\left(\frac{{OD\left({compound}_{X}\right)}_{595,48h}-{OD\left({compound}_{X}\right)}_{595,24h}}{{OD\left(control\right)}_{595,48h}-{OD\left(control\right)}_{595,24h}}*100\right)$$

Equation (1): Calculation of growth inhibition percentage.

### 2.7. Determination of Minimal Inhibitory Concentration (MIC)

Based on the results of the agar disc diffusion method and the screening in liquid medium, eight compounds were selected for further testing. The minimal inhibitory concentration of octanoic acid, allyl isothiocyanate, hexanoic acid, 2,3-butanedione, E-cinnamaldehyde, carvacrol, acetaldehyde and benzyl isothiocyanate was determined against *A. westerdijkiae*, *P. hordei* and *P. palitans*. These three species were selected based on their prevalence in spoiled par-baked bread and phylogenetic distance to ensure the selection of compounds with broad-spectrum activity [8]. The method for the determination of the MIC was based on the protocol published by Eucast [30], with some slight modifications. For each compound, a two-fold dilution series starting from 1024 µg/mL was tested. First, a two-fold dilution series was prepared in microcentrifuge tubes, using ethanol as a diluent. Then, the compound was further diluted using semi-solid YES medium to twice the desired final concentration, and 100 µL was added to a 96-well plate. The wells with 0 µg/mL of the tested compound contained 100 µL semi-solid YES medium.

Then, 100 µL semi-solid YES medium inoculated with 10,000 spores/mL was added to obtain a final concentration of 1000 spores per well. The outer wells of the 96-well plates were filled with 200 µL semi-solid YES medium to minimize evaporation of the inner wells. The plates were sealed using a Breathe-Easy^®^ film (Sigma-Aldrich) and incubated for 48 h at 26 °C. Every 24 h, the optical density (OD) was measured at 595 nm. For each compound, two biological replicates, each consisting of three technical replicates, were included against each mould species. Antifungal activity was expressed as the mean relative percentage of growth inhibition of the six technical replicates, compared to the growth in absence of a compound. The minimal inhibitory concentration was determined as the minimal concentration needed to obtain 90% growth inhibition (MIC_90_).

### 2.8. Synergy Testing: Checkerboard Assay

To study possible synergies between compounds, the checkerboard assay was performed. The protocol was based on the paper published by Bellio et al. [31], with some adaptations. Combinations of octanoic acid, allyl isothiocyanate, hexanoic acid, 2,3-butanedione, E-cinnamaldehyde, carvacrol, acetaldehyde and benzyl isothiocyanate were tested against *A. westerdijkiae*, *P. hordei* and *P. palitans*. First, stock solutions of the two compounds were made, based on the previously determined MIC_90_. Of compound 1, a solution of 4× MIC_90_ and 8× MIC_90_ was made, as well as a 4× MIC_90_ solution of compound 2. The outer wells of the 96-well plates were filled with 200 µL semi-solid YES medium to minimize evaporation of the inner wells. To the inner wells, 100 µL of semi-solid YES medium was added. Then, 100 µL of the 4× MIC_90_ solution of compound 1 was added to well B2 to B10, while 100 µL of the 8× MIC_90_ solution was added to well B11. Compound 1 was two-fold diluted from row B to row F using a multichannel pipette by transferring 100 µL to each executive row and discarding 100 µL after reaching row F. Then, 100 µL of the 4× MIC_90_ solution of compound 2 was added to wells B11 to G11, and compound 2 was two-fold diluted from column 11 to 3 by transferring 100 µL to each executive column. Again, 100 µL was discarded after reaching column 3. Finally, 100 µL of inoculated semi-solid YES medium was added to each inner well, resulting in a final volume of 200 µL and 1000 spores in each well. The plates were sealed using a Breathe-Easy film (Sigma-Aldrich) and incubated for 48 h at 26 °C. Every 24 h, the optical density (OD) was measured at 595 nm. For each combination, at least one biological replicate with three technical replicates was included. If a potentially interesting synergistic interaction was observed, the experiment was repeated with a second biological replicate, also including three technical replicates. This was the case for acetaldehyde combined with either benzyl isothiocyanate, 2,3-butanedione or carvacrol, benzyl isothiocyanate combined with 2,3-butanedione, carvacrol or octanoic acid, 2,3-butanedione combined with carvacrol, hexanoic acid or octanoic acid, carvacrol combined with allyl isothiocyanate, and E-cinnamaldehyde combined with allyl isothiocyanate. The synergistic potency was calculated based on the MuSyC principle [32] by using the average relative growth of three or six technical replicates after 48 h. The synergistic potency (α) indicates the change in potency of one compound in the presence of the other compound. If the 95% confidence interval (CI) includes 1, the effect is indifferent. When all values of the 95% CI are greater or less than 1, it indicates synergistic or antagonistic potency, respectively.

### 2.9. Validation in Bread Matrix

The effect of benzyl isothiocyanate and carvacrol was further validated in par-baked bread. In case of the shelf life test, determination of colour parameters and sensorial validation, the breads were surface treated with the compounds using an airbrush system and packaged under modified atmosphere. The challenge test included an additional step in which the surface was inoculated with mould spores in between the surface treatment with the compounds and packaging. Calcium propionate served as a positive control and was added to the dough instead of spray application.

#### 2.9.1. Bread-Making Procedure

All experiments were performed using a single batch of commercial wheat flour EPI B type 55 (Paniflower, Merksem, Belgium). Water absorption and malt falling number were experimentally determined using Farinograph-E (Brabender, Duisburg, Germany) and were 58.9% and 0.27%, respectively. A total of 100 g of flour, 58.9 g of water, 1.5 g of table salt, 0.27 g of malt flour, 1 g of instant dry baker’s yeast (Algist Bruggeman, Ghent, Belgium) and 0.005 g of ascorbic acid was weighed, and the ingredients were mixed for six minutes in a De Danieli spiral mixer (Verhoest Machinery, Izegem, Belgium). To obtain breads with 0.15% propionic acid, 0.63 g calcium propionate (Sigma-Aldrich) per 100 g flour was added as well.

After mixing, the dough was placed in a proving cabinet (Panimatic, Souppes-sur-Loing, France) for ten minutes at 30 °C and 80% to 90% relative humidity. After ten minutes, the dough was divided into pieces of 65 g (±1 g) and shaped manually. The dough pieces were then placed on a perforated plate, greased to prevent the dough from sticking, and placed in the proving cabinet (Panimatic) for 60 min at 30 °C and 80% to 90% relative humidity. Then, the dough pieces were baked in the oven (MIWE Aeromat FB12, type 4.64) in two phases. The first phase consisted of two minutes of baking at 170 °C and 200 mL steam injection, in which the steam valve was closed. In the second phase, the steam valve was open and there was no steam injection. This phase consisted of eight minutes of baking at 150 °C. The par-baked breads were cooled to room temperature and then transported in sterile bags.

#### 2.9.2. Treatment and Packaging

Based on the results of the MIC and checkerboard assay, different concentrations of benzyl isothiocyanate and carvacrol were tested. Table 1 summarizes all combinations of concentrations of the active compounds in the spraying solution that were tested. In both the shelf life and challenge tests, ten technical repeats were included for each combination of concentrations. Calcium propionate was included as a positive control, containing fourteen technical repeats, while par-baked breads without treatment were included as a negative control (T0), containing twelve technical repeats. As the compounds were diluted in ethanol, a control with ethanol was included as well (T1) with ten technical repeats. The compounds were sprayed on the surface of the par-baked bread using an airbrush system, with a total of 1 mL sprayed on each par-baked bread, holding the airbrush system at a distance of 15 cm, moving it up and down, while rotating the sample. After a full rotation, the samples were placed two per PP/EVOH/PP (PP: polypropylene, EVOH: ethylene vinylalcohol) transparent tray (Deca Pack) and packaged under modified atmosphere using a Tray Sealer (DECA Packaging Group, Herentals, Belgium) at a gas composition of 50% CO_2_ and 50% N_2_, using a OPA/PE/EVOH/PE/PP (OPA: orientated polyamide, PE: polyethylene) cover film (Opalen HB 65 AF peel 430 mm, Amcor). In case of the challenge test, the surface of the par-baked breads was inoculated with mould spores before packaging.

#### 2.9.3. Antifungal Validation in Bread Matrix

To determine the antifungal effect of benzyl isothiocyanate and carvacrol in the bread matrix, both a shelf life test and challenge test were performed. In case of the shelf life test, the par-baked breads were packaged after treatment and stored for 30 days at 22 °C. Visible mould growth was checked every other day. In the challenge test, a spot containing 200 spores/10 µL of *P. palitans* was applied in the centre of the bread’s surface after spraying and right before packaging. Similar to the shelf life test, the samples were stored for 30 days at 22 °C and checked for visible mould growth every other day. In case of the challenge test, only growth due to inoculation of spores was taken into account and not mould growth due to environmental contamination. To assess differences in antifungal activity between two treatments, statistical analysis was performed using GraphPad Prism version 10.5.0 (GraphPad Software, San Diego, CA, USA). Normality was checked by performing a Shapiro–Wilk test. In cases of normal distribution, an unpaired *t* test was performed, while a Mann–Whitney U test was used when data was not normally distributed.

#### 2.9.4. Determination of Colour Parameters

After two days of storage at 22 °C, the par-baked breads were fully baked in two phases. The first phase consisted of two minutes at 220 °C and 200 mL steam, while the second phase was eight minutes of baking at 200 °C. The CM700d/600d spectrophotometer (Konica Minolta) was used to the determine the crust’s colour parameters (lightness (L*), green–red axis (a*), blue–yellow axis (b*)), standardized with a white calibration plate. Colour parameters were measured of samples containing 300 µg/mL benzyl isothiocyanate and 1000 µg/mL carvacrol, based on the results of the shelf life and challenge test, untreated samples and samples containing calcium propionate. Two biological repeats were included for the treated samples, while four biological repeats were included for the untreated samples and samples containing calcium propionate. To assess differences in colour parameters between two treatments, statistical analysis was performed using GraphPad Prism version 10.5.0 (GraphPad Software, San Diego, CA, USA). Normality was checked by performing a Shapiro–Wilk test. In cases of normal distribution, an unpaired *t* test was performed, while a Mann–Whitney U test was used when data was not normally distributed.

#### 2.9.5. Sensorial Validation in Bread Matrix

Based on the results of the shelf life and challenge tests, it was decided to investigate the effect of 300 µg/mL benzyl isothiocyanate and 1000 µg/mL carvacrol on the sensorial quality of par-baked bread by performing a triangle test. In this test, each participant received three samples of fully baked bread, in which two samples were identical and one sample was different. Water was provided throughout the test. Combinations and order of untreated par-baked bread and treated par-baked bread were randomized using EyeQuestion 5.4.7 software (Logic8 BV, Elst, The Netherlands). The test was carried out in the sensory lab facilities (SensoLab) of Ghent University and included 91 participants. These were employees and students present at the faculty of Bioscience Engineering (Campus Coupure, UGent) and who had no former experience in sensory tasting. Every participant was asked whether they tasted a difference in the samples and to indicate the different sample. In case no difference was tasted, participants were obliged to guess which sample was different. Statistical analysis was carried out using EyeQuestion 5.4.7 software (Logic8 BV, Elst, The Netherlands) by performing a binomial test.

## 3. Results

### 3.1. Antifungal Activity of Plant Secondary Compounds

As part of the plant defence mechanism, plant secondary metabolites (PSMs) are considered as a valuable source for natural antimicrobials. In this study, 218 compounds were screened for their activity against ten bread moulds collected from spoiled par-baked bread. Each compound was evaluated for its growth-inhibiting effect in an agar disc diffusion assay and an antifungal susceptibility test in liquid medium.

**Figure 1:**
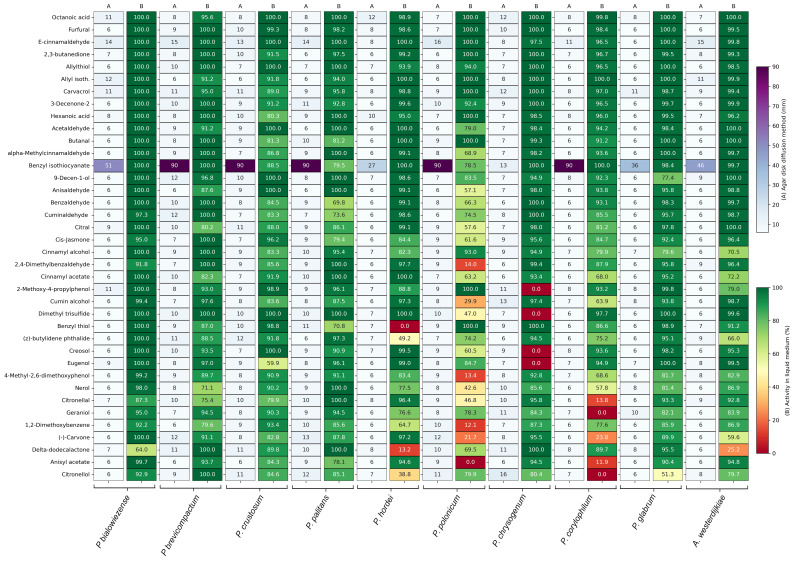
Antifungal activity of the best-performing plant secondary metabolites against ten bread moulds, with (**A**) the inhibition zone in mm in the agar disc diffusion assay and (**B**) the percentage growth inhibition, relative to untreated spores, in the antifungal susceptibility test. For each compound, two technical replicates were included against each mould strain. Growth inhibition is the average of two technical repeats.

In the agar disc diffusion assay, inoculated plates were exposed to a filter disc containing 5 µL of a 1% compound solution. The inhibition zone was measured after 48 h of incubation. The inhibition zones of the best-performing compounds are displayed in Figure 1A and are expressed in mm. Supplementary Table S2A contains the inhibition zones of all 218 tested compounds. The majority of the compounds had an inhibition zone of 6 mm and were considered as non-inhibitory, as the inhibition zone did not exceed the diameter of the filter disc. Benzyl isothiocyanate had the strongest antifungal activity in the agar disc diffusion assay, with complete inhibition against *Penicillium brevicompactum*, *P. crustosum*, *P. palitans*, *P. polonicum* and *P. corylophilum*. It was the only compound in the screening collection capable of completely inhibiting fungal growth at the tested concentration. It also showed strong inhibition against *P. bialowiezense* (51 mm), *Aspergillus westerdijkiae* (46 mm), *P. glabrum* (36 mm) and *P. hordei* (37 mm). *Penicillium chrysogenum* was the most resistant to benzyl isothiocyanate, with an inhibition zone of 13 mm. Although less strong than benzyl isothiocyanate, E-cinnamaldehyde also showed broad-spectrum activity, inhibiting the growth of six of the tested species with an inhibition zone of 12 mm or greater.

The agar disc diffusion assay is a good method to test a large collection of compounds, as it is simple and low in cost; however, due to its qualitive and not quantitative nature, it does not allow minimal inhibitory concentration (MIC) determination [33]. Moreover, the compound’s concentration within the agar is dependent on the compound’s characteristics, including the molecular weight, solubility and the diffusion rate [34]. As plant secondary metabolites, and especially essential oil compounds, are more hydrophobic compounds, these compounds will not easily diffuse throughout the water-based agar. Therefore, we performed an antifungal susceptibility test in liquid medium in parallel.

When testing the antifungal activity in liquid medium, all compounds were tested against the ten bread moulds at one fixed concentration, 500 µg/mL, and evaluated for their ability to inhibit growth after 48 h of incubation. Figure 1B summarizes the percentage of relative growth inhibition of the best-performing compounds, while Supplementary Table S2B contains the percentage relative growth inhibition of the entire plant secondary metabolite collection. More compounds were able to inhibit mould growth in the liquid medium compared to the agar disc diffusion assay. Of the 218 compounds tested against ten moulds, total growth inhibition was reached 5 times (0.23%) in the agar disc diffusion assay and 125 times (5.73%) in the screening in liquid medium. Besides benzyl isothiocyanate, many plant secondary metabolites showed weak or no inhibition in the agar disc diffusion assay, while being good to strong inhibitors in the liquid medium, with 14.31% and 19.27% of the compounds having relative growth inhibition above 90% and 80%, respectively.

The best-performing compounds in the antifungal susceptibility test in liquid medium were dominated by aldehydes, followed by alcohols, acids, isothiocyanates, phenols and ketones. Octanoic acid, furfural and E-cinnamaldehyde had the best broad-spectrum activity, with a relative growth inhibition of 95% or higher against all tested species. Based on these results, octanoic acid, E-cinnamaldehyde, 2,3-butanedione, allyl isothiocyanate, carvacrol, hexanoic acid, acetaldehyde and benzyl isothiocyanate were selected for further testing.

### 3.2. Minimal Inhibitory Concentration of Selected PSM

**Figure 2:**
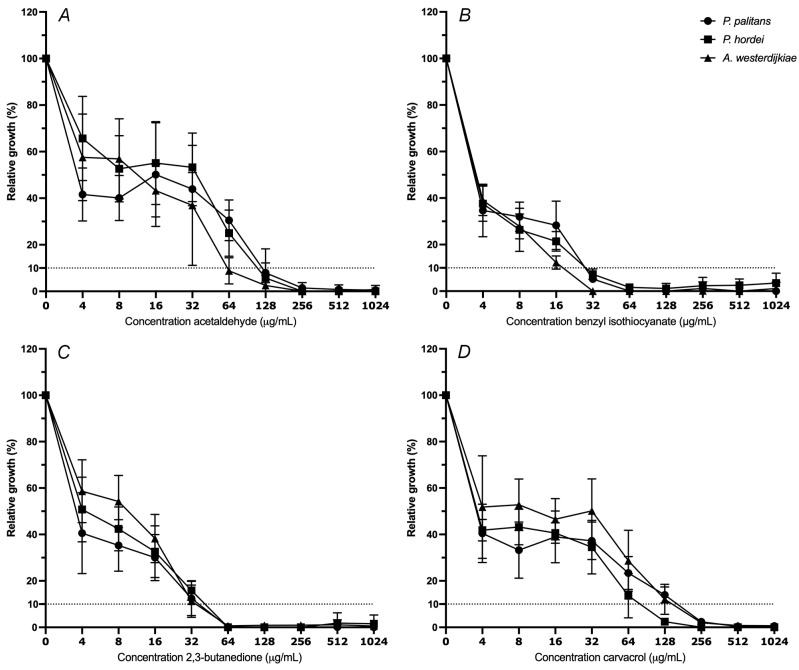
Relative growth (%) of *Penicillium palitans* (●), *P. hordei* (■) and *Aspergillus westerdijkiae* (▲) at increasing concentrations (µg/mL) of acetaldehyde (**A**), benzyl isothiocyanate (**B**), 2,3-butanedione (**C**) and carvacrol (**D**). Each experiment contained three technical repeats and was repeated twice. Growth is relative to the condition containing no treatment (0 µg/mL) and is the average of six repeats. The dotted line indicates 10% of relative growth.

The minimal inhibitory concentration (MIC) of octanoic acid, E-cinnamaldehyde, 2,3-butanedione, allyl isothiocyanate, carvacrol, hexanoic acid, acetaldehyde and benzyl isothiocyanate was determined against *P. palitans*, *P. hordei* and *A. westerdijkiae*. The MIC at 90% of relative growth inhibition after 48 h of incubation was determined. The moulds were exposed to a ½ dilution series starting from 1024 µg/mL. The decrease in percentage of relative growth with increasing concentration of acetaldehyde (A), benzyl isothiocyanate (B), 2,3-butanedione (C) and carvacrol (D) is reported in Figure 2. The MIC_90_ of octanoic acid, allyl isothiocyanate, hexanoic acid and E-cinnamaldehyde can be found in Supplementary Table S3.

The isothiocyanates exerted the strongest antifungal activity. MIC_90_ of *A. westerdijkiae*, *P. palitans* and *P. hordei* was obtained at 16, 16 and 32 µg/mL allyl isothiocyanate, respectively, while 32 µg/mL of benzyl isothiocyanate was required for 90% growth inhibition of all tested species. At 64 µg/mL, the two aldehydes and ketone—acetaldehyde, E-cinnamaldehyde and 2,3-butanedione—all had the same inhibiting effect on *A. westerdijkiae*. *Penicillium palitans* was slightly more resistant to acetaldehyde, and E-cinnamaldehyde and *P. hordei* to acetaldehyde, requiring 128 µg/mL to obtain 90% growth inhibition. In case of carvacrol, a reduction of fifty percent of growth was already achieved at 4 µg/mL; however, further reduction to 90% growth inhibition was obtained at 128 µg/mL for *P. hordei* and 256 µg/mL for *P. palitans* and *A. westerdijkiae*. Together with carvacrol, hexanoic acid and octanoic acid were the least active of the tested compounds.

### 3.3. Synergy of Selected PSMs

By performing checkerboard assays, combinations of octanoic acid, E-cinnamaldehyde, 2,3-butanedione, allyl isothiocyanate, carvacrol, hexanoic acid, acetaldehyde and benzyl isothiocyanate were tested for synergistic interactions against *P. palitans*, *P. hordei* and *A. westerdijkiae*. We calculated the synergistic potency based on the MuSyC principle [32] by using the relative growth after 48 h. The synergistic potency (α) indicates the change in potency of one compound in the presence of the other compound. An indifferent effect is found when the 95% confidence interval (CI) includes 1. When all values of the 95% CI are greater or less than 1, it indicates synergistic or antagonistic potency, respectively.

**Figure 3:**
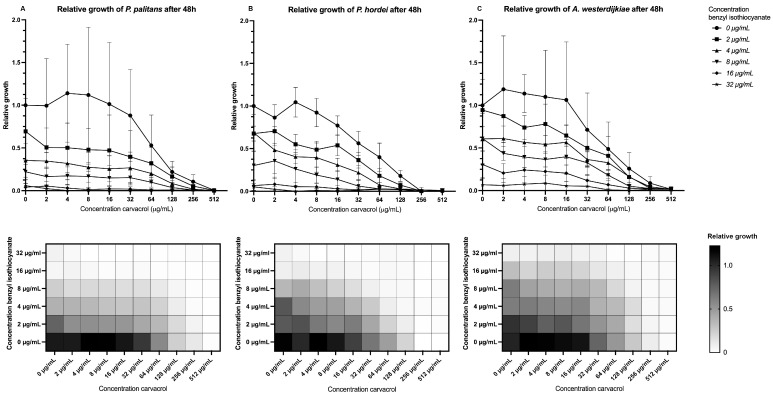
Relative growth of (**A**) *P. palitans*, (**B**) *P. hordei* and (**C**) *A. westerdijkiae* at different concentrations of carvacrol and benzyl isothiocyanate after 48 h of incubation. Each experiment contained three technical repeats and was repeated twice. Growth is relative to the condition containing no treatment (0 µg/mL) and is the average of six repeats.

When combining benzyl isothiocyanate with carvacrol, a relative growth below 0.1, or growth inhibition of 90%, was found when combining 8 µg/mL benzyl isothiocyanate and 64 µg/mL carvacrol for *P. palitans* (Figure 3A), 8 µg/mL benzyl isothiocyanate and 32 µg/mL carvacrol for *P. hordei* (Figure 3B), and 16 µg/mL benzyl isothiocyanate and 64 µg/mL carvacrol for *A. westerdijkiae* (Figure 3C). This effect was confirmed using the MuSyc framework [32], and we found that benzyl isothiocyanate had a positive effect on carvacrol by increasing the latter’s potency with a magnitude of 131.8 and 15.22 against *P. palitans* and *P. hordei*, respectively. An additive effect was found against *A. westerdijkiae*. Carvacrol had a synergistic effect on benzyl isothiocyanate’s potency against *P. hordei* and an additive effect against *P. palitans* and *A. westerdijkiae*.

**Figure 4:**
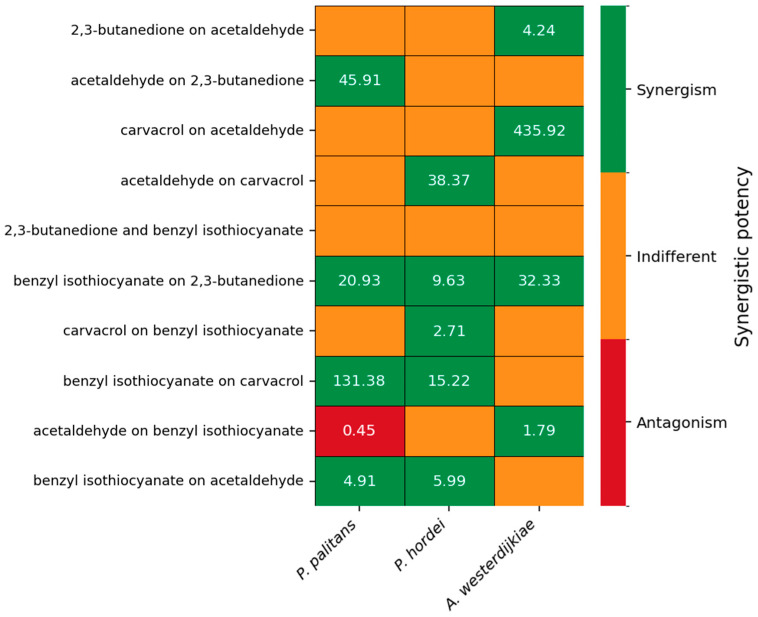
Synergistic potency of 2,3-butanedione and acetaldehyde, carvacrol and acetaldehyde, 2,3-butanedione and benzyl isothiocyanate, carvacrol and benzyl isothiocyanate, and acetaldehyde and benzyl isothiocyanate against *P. palitans* (left), *P. hordei* (middle) and *A. westerdijkiae* (right). Each experiment contained three technical repeats and was repeated twice. Synergistic potency is calculated based on the MuSyc principle [32], using the average relative growth of six repeats. The synergistic potency (α) indicates the change in potency of one compound in the presence of the other compound. If the 95% confidence interval (CI) includes 1, the effect is indifferent (orange). When all values of the 95% CI are greater or less than 1, it indicates synergistic (green) or antagonistic (red) potency, respectively.

The synergistic potency of the five most interesting combinations is depicted in Figure 4. Synergistic potency and the confidence interval of all combinations can be found in Supplementary Table S4. Benzyl isothiocyanate was able to increase the potency of 2,3-butanedione against *P. palitans*, *P. hordei* and *A. westerdijkiae*. When combining aldehydes with carvacrol, synergistic interactions occurred as well. 2,3-butanedione increased carvacrol’s potency against *P. palitans* and *P. hordei*; however, 2,3-butanedione’s activity was negatively affected by carvacrol when tested against *P. hordei*. Acetaldehyde improved the activity of carvacrol against *P. hordei* while carvacrol had a positive effect on acetaldehyde against *A. westerdijkiae*. Combinations with hexanoic acid, octanoic acid or allyl isothiocyanate resulted in no synergy or synergy against only one of the three tested species.

It was decided to further validate the antifungal activity of benzyl isothiocyanate and carvacrol in a par-baked bread matrix. Essential oils rich in these compounds are widely available, making industrial application of these compounds, or essential oils rich in these compounds, more feasible. Benzyl isothiocyanate can be found in plants of the Brassicaceae family, while carvacrol is a major component in essential oils obtained from plants of the genus *Thymus*, *Satureja* and *Origanum* [35,36].

### 3.4. Validation of Benzyl Isothiocyanate and Carvacrol in a Bread Matrix

#### 3.4.1. Impact on the Shelf Life of Par-Baked Bread

**Figure 5:**
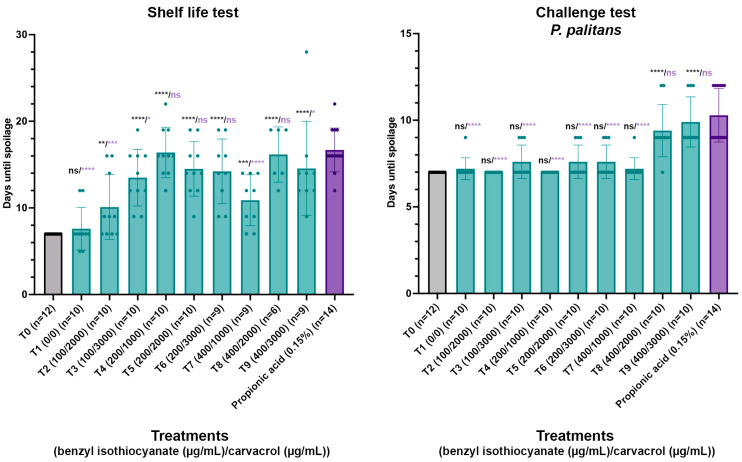
Shelf life and challenge test with *P. palitans* with no treatment (T0, n = 12), treatment with ethanol (T1, n = 10), combinations of benzyl isothiocyanate (µg/mL) and carvacrol (µg/mL) (T2–T9, n = 10) and propionic acid (0.15%, n = 14). Par-baked bread was surface sprayed using an airbrush system, packaged under modified atmosphere and stored at 22 °C. Samples were checked daily for visible mould growth. Bars and error bars represent the mean and standard deviation, respectively. Dots indicate individual samples. Statistical analysis was performed between treated samples and either untreated samples or samples containing calcium propionate, with significance level shown in black or purple, respectively. (* *p* ≤ 0.05, ** *p* ≤ 0.01, *** *p* ≤ 0.001, **** *p* ≤ 0.0001, ns *p* > 0.05).

Based on the results obtained in the MIC and checkerboard assays, different combinations of concentrations of benzyl isothiocyanate and carvacrol were validated for the antifungal activity in a par-baked bread matrix. A total of eight combinations were tested (T2–T9), going from 100 µg/mL to 400 µg/mL benzyl isothiocyanate combined with 2000 µg/mL to 3000 µg/mL carvacrol in a shelf life and challenge test, and these treatments all contained ten repeats. A control with no treatment (T0) and a control containing 0.15% propionic acid were included, with twelve and fourteen repeats, respectively. As the compounds were dissolved in ethanol, a control containing only ethanol (T1) was included as well, containing ten repeats. The compounds were sprayed on the surface of par-baked bread using an airbrush system and packaged under modified atmosphere using a gas mixture of 50% CO_2_ and 50% N_2_. In the challenge tests, spores of *P. palitans* were applied on the bread surface between spraying and packaging. In previous research, spoilage of par-baked bread was dominated by *P. palitans* [8]. After packaging under modified atmosphere, the samples were stored at 22 °C and followed daily for visible mould growth. The days until spoilage for each condition can be found in Figure 5, and the performance of each treatment is statistically compared to the condition with no treatment and the condition containing 0.15% propionic acid.

In both the shelf life test and challenge tests, ethanol had no significant effect compared to no treatment. In the shelf life test, all treatments worked significantly better than no treatment, while T4, T5, T6 and T8 worked as good as 0.15% propionic acid. Furthermore, not all samples of T6, T7, T8 and T9 were spoiled within 30 days incubation, while all samples with propionic acid were spoiled within 22 days since packaging. The uncontaminated samples were kept and monitored daily until the packaging collapsed. As these samples remained free from visible mould, it appears that treatments T6, T7, T8 and T9 worked even better than 0.15% propionic acid. However, the shelf life test relies on environmental contamination and results in great variability throughout the test, which makes it difficult to draw sound conclusions. The challenge test serves as a good alternative to diminish this variability by inoculating the bread surface with a known number of spores and allows us to observe the response in a worst-case scenario where all samples are contaminated. In the challenge test, T8 and T9 acted significantly better than no treatment and as good as propionic acid (0.15%).

Application of 200 to 400 µg/mL benzyl isothiocyanate and 1000 to 2000 µg/mL carvacrol significantly increased the time until visible spoilage compared to untreated samples in both the shelf life and challenge tests while performing comparably to 0.15% propionic acid. Based on these results, it was decided to validate the impact of the spray application of 300 µg/mL benzyl isothiocyanate and 1000 µg/mL carvacrol on the colour parameters and sensorial quality of the par-baked bread.

#### 3.4.2. Impact on the Colour Parameters of Par-Baked Bread

To assess the impact of the compounds on the colour parameters of the bread’s surface, 300 µg/mL benzyl isothiocyanate and 1000 µg/mL carvacrol were applied on par-baked bread through spray application. Then, the samples were packaged under modified atmosphere and fully baked after two days of storage at 22 °C. Application of 300 µg/mL benzyl isothiocyanate and 1000 µg/mL carvacrol did not significantly affect the colour parameters, compared to untreated samples and samples treated with propionic acid. Table 2 lists the average L*, a* and b* values and the standard deviation.

#### 3.4.3. Impact on the Sensorial Quality of Par-Baked Bread

Sensorial impact was evaluated by performing a triangle test with treated and untreated par-baked bread. The treated par-baked bread contained 300 µg/mL benzyl isothiocyanate and 1000 µg/mL carvacrol, applied through spray application. Both treated and untreated samples were packaged under modified atmosphere and fully baked after two days of storage at 22 °C. Of the 91 participants, 44 participants correctly indicated the different sample. When alpha, the risk of concluding that a difference exists while the products are the same is below 0.05, no more than 39 participants can indicate the correct answer, meaning that our treated and untreated samples are significantly different. Some participants reported perceiving a slight difference in saltiness or a more pronounced herbal taste. Despite these minor sensory differences, the treatment was generally well accepted.

## 4. Discussion

To reduce food waste and improve food safety, preservatives have become an essential value in our daily lifestyle. In the search for a natural alternative for the currently used synthetic preservatives, we evaluated the effect of 218 plant secondary metabolites (PSM) against ten bread moulds isolated from spoiled par-baked bread. By including ten different mould species, we were able to screen for compounds active against multiple species instead of being limited to one or a few organisms. The antifungal activity in both agar-based and liquid-based methods was evaluated and synergistic interactions between the most promising compounds were investigated. The antifungal activity of benzyl isothiocyanate combined with carvacrol was further validated in par-baked bread through spray application. In addition, the impact of these compounds on the colour parameters and sensorial quality of fully baked bread was assessed.

### 4.1. Comparison of Disc Diffusion and Microdilution Assay

We implemented two different screening methods, with each method having its advantages and disadvantages. The agar disc diffusion assay is simple and does not require specialized equipment. It was used as a primary test to distinguish between compounds with and without antifungal activity but does not produce quantitative data, and the assessment of the inhibition zone may be subjective. Moreover, factors such as temperature, pH and humidity affect the rate of diffusion [34]. Many of the plant secondary metabolites have a more hydrophobic nature, reducing their ability to diffuse throughout the water-based agar medium. Using a diffusion method may underestimate the antifungal activity, and evaluation based on solely a diffusion method should be performed with great care [37]. By including an antifungal susceptibility test, we prevented overlooking interesting antifungal compounds. The test in liquid medium allows quantitative determination of the antifungal activity and automated read outs via optical density measurements. However, this method requires more material and access to a spectrophotometer [34].

Benzyl isothiocyanate and E-cinnamaldehyde were the only compounds with interesting antifungal activity in the agar disc diffusion assay. By comparing the agar diffusion and vapour assay, Inouye et al. found that oils rich in aldehydes performed better because of their capacity to diffuse through the agar, while oils with alcohols, ketones, esters, oxides and hydrocarbons were more active in the vapour phase [37]. This could explain the higher activity of E-cinnamaldehyde, although the other aldehydes lacked antifungal activity in this assay. The strong antifungal activity of benzyl isothiocyanate could possibly be explained by its volatile character. Inouye et al. hypothesized that the antifungal activity of hydrophobic compounds could be the result of redeposition of the compounds on the agar surface, thereby possibly explaining the complete inhibition by benzyl isothiocyanate [37]. By conducting vapour phase-based assays, the activity of these compounds in the vapour phase can be further elucidated.

Octanoic acid and hexanoic acid showed broad-spectrum activity in the screening in liquid medium and were the best- and ninth-best-performing compounds. Besides hexanoic and octanoic acid, other fatty acids were tested in the screening as well, including butyric acid, decanoic acid, dodecanoic acid, tetradecanoic acid, hexadecenoic acid and octadecanoic acid. The good activity of octanoic acid and hexanoic acid compared to other tested fatty acids in the screening was likely due to an optimal balance between hydrophobicity and solubility. As chain length increases, the compounds become more hydrophobic, allowing interaction with the lipid bilayer; however, as solubility decreases, the fatty acids form self-aggregated clusters [38]. Aldehydes and phenols also exhibited strong inhibition in the liquid screening method, which is similar to what is reported in the literature [39,40,41,42,43]. It is believed that the carbonyl group of aldehydes can bind to proteins and prevent amino acid decarboxylase activity [44], while phenols affect the proton gradient and motive force by delocalisation of electrons [18].

Overall, we could not compare antifungal activity between the two methods, as most compounds did not affect fungal growth in the agar disc diffusion assay while having varying effects in the microdilution assay. The literature agrees on the weak correlation between dilution and diffusion methods [45], while correlation between two diffusion methods [45] or between two dilution assays [46] was high. Considering that the more hydrophobic character of the plant secondary metabolites caused underestimation of their antifungal effect, the antifungal susceptibility test in liquid medium was preferred.

### 4.2. Synergistic Relation Between Plant Secondary Metabolites

The screening of PSMs revealed which compounds exerted interesting antifungal activity. However, to guarantee food safety, a single PSM needs to be added in such a high concentration that it affects the organoleptic properties of the product. Therefore, the synergistic interactions between octanoic acid, E-cinnamaldehyde, 2,3-butanedione, allyl isothiocyanate, carvacrol, hexanoic acid, acetaldehyde and benzyl isothiocyanate were investigated. Synergy can result from the complementary actions of two compounds on multiple targets within a biochemical pathway, their simultaneous action on the cell membrane, or the interaction of one compound enhancing the uptake of the other compound by increasing membrane permeability or decreasing drug efflux [20,21].

Isothiocyanates can be found in plants from the Brassicaceae family and are formed by enzymatic cleavage of glucosinolates upon plant damage [47]. Their broad-spectrum and strong activity [47,48,49] make them interesting antimicrobial compounds, which was also reflected in our screening. Benzyl isothiocyanate was the only compound with strong antifungal activity in the agar disc diffusion assay, and its volatile antifungal activity has been exploited for possible active packaging [50]. The MIC_90_ of the isothiocyanates was the lowest of all compounds tested in this research, and we found interesting synergistic interaction when combining benzyl isothiocyanate with carvacrol or 2,3-butanedione. It has been found that isothiocyanates do not affect the cell membrane and the membrane permeability directly but rather act intracellularly, thereby affecting the energy metabolism [49]. Due to their functional group, it is believed that isothiocyanates interact with the cysteine disulfide bond in proteins, inactivating intracellular enzymes [19]. The main constituent of oregano essential oil is carvacrol. Carvacrol is synthesized via the mevalonate pathway and has many other functions besides antimicrobial activity, such as antioxidant, insecticidal and anti-inflammatory actions [51]. Its antimicrobial activity is linked to its amphipathic character and removal of one of its substituents causes a decrease in antimicrobial activity [18]. The aliphatic side chain allows interaction of carvacrol with the membrane, while the hydroxyl group can act as a proton exchanger [17]. Undissociated carvacrol will diffuse through the membrane and releases a proton into the cytoplasm. Dissociated carvacrol binds a cation and returns to the extracellular space. This will disrupt the proton motive force and affect ATP synthesis [17]. In addition, the accumulation of carvacrol in the cell membrane may alter the arrangement and stability of the phospholipid bilayer, thereby increasing membrane permeability [52]. Membrane permeabilization by carvacrol may facilitate the uptake of benzyl isothiocyanate, which subsequently inactivates intracellular enzymes. In addition, the synergistic interaction may result from both compounds affecting energy metabolism, with carvacrol disrupting the electrochemical gradient and benzyl isothiocyanate altering the structure and function of proteins involved in energy metabolism [17,19]. However, a small interaction between benzyl isothiocyanate and E-cinnamaldehyde was observed in this study, while the latter is believed to affect energy metabolism as well by inhibiting ATPase [53]. 2,3-butanedione, on the other hand, targets the cell membrane, resulting in increased permeability and leakage of ions. Shi et al. proposed this was mediated via induced reactive oxygen species (ROS) accumulation [54]. This may explain the synergy found between 2,3-butanedione and benzyl isothiocyanate; as 2,3-butanedione increases the permeability of the cell membrane, the entrance of benzyl isothiocyanate into the cell could increase, thereby enhancing its effectiveness.

During the screening, octanoic acid and hexanoic acid showed great potential with activity against all ten tested bread mould strains. By inserting in the lipid bilayer, fatty acids disturb the fungal cell membrane and increase membrane fluidity. This causes conformational changes in membrane proteins and release of intracellular components, resulting in cell death [55]. We hypothesized that the disruption of the plasma membrane, would have facilitated the uptake of the combined compound; however, no interesting synergy was found in the combination that included either hexanoic or octanoic acid. The insertion into the lipid bilayer, and thus their bioactivity, is affected by the surrounding pH. At a lower pH, fatty acids are in a more undissociated state, making it easier to penetrate the membrane. The pH of semi-solid YES medium was 6.5, impacting the final efficacy. Lowering the pH of the surrounding medium could increase the fatty acids activity and might facilitate synergy with other compounds. Additional experiments can be conducted to investigate this pH-dependent effect.

### 4.3. Validation of Benzyl Isothiocyanate and Carvacrol in a Par-Baked Bread Matrix

The in vitro antifungal activity of benzyl isothiocyanate combined with carvacrol was validated through spray application on a par-baked bread matrix by performing shelf life and challenge tests. The combined results of the shelf life test and challenge test indicated that at least 200 to 400 µg/mL benzyl isothiocyanate and 1000 to 2000 µg/mL carvacrol is needed to inhibit mould growth in par-baked bread and act as a good replacement of the current strategy with propionic acid. When validating in vitro results in a food matrix, the literature reports a decrease in antifungal activity [22,24,56,57,58,59,60]. Components present in the food matrix, as well as extrinsic factors such as temperature, packaging and application method, can affect the compound’s antimicrobial efficiency. As a result, it is often found that a higher concentration of the antimicrobial compound is needed, thereby affecting the organoleptic properties of the product [24]. In general, it is found that the efficacy of essential oil (compounds) increases as pH, fat content and amount of carbohydrates decreases, and also as protein levels increases [61]. Debonne et al. validated the antifungal effect of thyme essential oil by incorporating the oil in the dough of par-baked bread and found that five to seven times more oil needs to be incorporated to have a significant increase in shelf life [22]. Moreover, the essential oil impacted the bread’s volume and colour parameters [22,23]. By applying the compounds through spraying instead of incorporation in the dough, potential degradation and evaporation of the compounds during baking was avoided, as well as possible negative effect on the leavening capacities of baker’s yeast. Moreover, spray application reduces the required amount of the compound compared to incorporation in the dough and ensures a more homogenous application throughout the bread surface. On an industrial scale, spray application would require specialized equipment, including nozzle systems and a conveyor belt. In recent years, the industrial application of ethanol has gained considerable interest. By adapting such systems to include a formulation with benzyl isothiocyanate and carvacrol, the same production line could be used for the application of these natural antimicrobial compounds.

Application of 300 µg/mL benzyl isothiocyanate and 1000 µg/mL carvacrol did not significantly impacted the bread’s surface colour parameters. However, at these concentrations, spray application of benzyl isothiocyanate combined with carvacrol did affect the sensorial quality of the par-baked bread, giving it a more herbal flavour compared to untreated samples. Although not negatively affecting the taste of the par-baked bread, additional efforts can be made to further limit its impact on the par-baked bread, such as investigating the effect of encapsulation of the compounds. Encapsulation of antimicrobial compounds can enhance heat stability and reduce volatility, while also limiting degradation by light and oxygen [62,63]. Moreover, encapsulation allows a more gradual and controlled release, and several studies have reported increased antimicrobial activity of encapsulated essential oils and essential oil compounds [64,65]. However, the choice of encapsulation system and method can influence the particle’s efficacy and stability.

This study revealed the potential of the combined application of benzyl isothiocyanate and carvacrol in par-baked bread. The applicability of these compounds should be further explored by optimizing the formulation and investigating the storage stability. Currently, benzyl isothiocyanate and carvacrol are taken up in the food flavouring database of EFSA and are allowed to be used in food if it improves the odour and/or taste of the product. However, when added for a technological purpose, such as antimicrobial preservation, the compounds should be treated as food additives.

## 5. Conclusions

Due to changing consumer behaviour towards products with synthetic preservatives, natural compounds with antifungal activity have attracted more and more interest. Plant secondary metabolites, and especially essential oils, offer a good solution; however, as the composition of these extracts are very variable, it is difficult to compare research findings. By screening a collection of 218 plant secondary compounds against bread moulds, we identified which compounds have interesting antifungal activity and compared the efficacy of agar-based and liquid-based screening methods. To increase their antimicrobial potency, the most interesting growth-inhibiting compounds were combined and investigated for synergy. Benzyl isothiocyanate combined with carvacrol was further validated in a par-baked bread matrix, investigating the antifungal efficacy in a shelf life test and challenge test. When applying 200 µg/mL of benzyl isothiocyanate and 1000 µg/mL carvacrol, an average shelf life of 16.4 days was reached, which was not significantly different from the shelf life of samples containing 0.15% propionic acid but significantly different from untreated samples. A similar result was found in the challenge test when applying 400 µg/mL of benzyl isothiocyanate and 2000 µg/mL carvacrol. Spray application of benzyl isothiocyanate and carvacrol had no influence on the colour of the par-baked samples but did impact the taste by giving it a more herbal aroma compared to untreated samples.

## Figures and Tables

**Table 1:** Combinations of concentrations of benzyl isothiocyanate and carvacrol in the spraying solutions tested in shelf life and challenge test. For each combination of concentrations, ten technical repeats were included in each test.

**Treatment**	**Benzyl Isothiocyanate (µg/mL)**	**Carvacrol (µg/mL)**
T2	1 00	2000
T3	100	3000
T4	200	1000
T5	200	2000
T6	200	3000
T7	400	1000
T8	400	2000
T9	400	3000

**Table 2:** Average lightness (L*), green–red axis (a*) and blue–yellow axis (b*) values and their standard deviation. Colour parameters of untreated samples, samples treated with propionic acid (0.15%) and treatment with benzyl isothiocyanate and carvacrol were determined of fully baked bread samples, cooled to room temperature. For each treatment, two technical repeats were included. No significant difference was found between the different treatments.

Treatment	L*	a*	b*
No treatment	64.85 ± 1.13	11.98 ± 1.24	30.77 ± 0.67
Propionic acid (0.15%)	66.67 ± 2.12	11.79 ± 1.84	31.48 ± 1.73
300 µg/mL benzyl isothiocyanate + 1000 µg/mL carvacrol	65.83 ± 0.08	12.80 ± 0.56	32.44 ± 0.04

## Data Availability

The original contributions presented in this study are included in the article and Supplementary Materials. Further inquiries can be directed to the corresponding author.

## Supplementary Materials

The following supporting information can be downloaded at: https://www.mdpi.com/article/10.3390/foods14213604/s1, Table S1: List of PSMs, Table S2: Antifungal activity of PSMs against ten bread moulds, Table S3: MIC_90_ of selected PSMs against *P. palitans*, *P. hordei* and *A. westerdijkiae*, Table S4: Synergistic potency of selected PSMs against *P. palitans*, *P. hordei* and *A. westerdijkiae*, Figure S1: Growth curves of the ten bread moulds.

## Abbreviations

The following abbreviations are used in this manuscript:

CI	Confidence interval
EFSA	European Food Safety Authority
MIC_90_	Minimal inhibitory concentrations, at 90% growth inhibition
PDA	Potato dextrose agar
PSM	Plant secondary metabolite
ROS	Reactive oxygen species
YES	Yeast extract sucrose

## References

[1] Almeida E.L., Steel C.J., Chang Y.K. (2016). Par-baked Bread Technology: Formulation and Process Studies to Improve Quality. Crit. Rev. Food Sci. Nutr..

[2] Melini V., Melini F. (2018). Strategies to Extend Bread and GF Bread Shelf-Life: From Sourdough to Antimicrobial Active Packaging and Nanotechnology. Fermentation.

[3] Alpers T., Kerpes R., Frioli M., Nobis A., Hoi K.I., Bach A., Jekle M., Becker T. (2021). Impact of Storing Condition on Staling and Microbial Spoilage Behavior of Bread and Their Contribution to Prevent Food Waste. Foods.

[4] Cornea C.P., Ciucǎ M., Voaides C., Gagiu V., Pop A. (2011). Incidence of fungal contamination in a Romanian bakery a molecular approach. Rom. Biotechnol. Lett..

[5] Lund F., Filtenbory O., Westall S., Frisvad J.C. (1996). Associated mycoflora of rye bread. Lett. Appl. Microbiol..

[6] Pitt J.I., Hocking A.D. (2009). Fungi and Food Spoilage.

[7] Dos Santos J.L.P., Bernardi A.O., Pozza Morassi L.L., Silva B.S., Copetti M.V., Sant’Ana A.S. (2016). Incidence, populations and diversity of fungi from raw materials, final products and air of processing environment of multigrain whole meal bread. Food Res. Int..

[8] Lepoutre A., Debonne E., Martini S., Devlieghere F., Van Dijck P. (2025). Isolation and identification of the mycobiome of spoiled par-baked bread produced in Western Europe. Microbe.

[9] Deschuyffeleer N., Audenaert K., Samapundo S., Ameye S., Eeckhout M., Devlieghere F. (2011). Identification and characterization of yeasts causing chalk mould defects on par-baked bread. Food Microbiol..

[10] Pundir R.K., Jain P. (2011). Qualitative and quantitative analysis of microflora of Indian bakery products. J. Argic. Technol..

[11] Khoshakhlagh K., Hamdami N., Shahedi M., Le-Bail A. (2014). Quality and microbial characteristics of part-baked Sangak bread packaged in modified atmosphere during storage. J. Cereal Sci..

[12] Erickson M.C., Doyle M.P. (2017). The Challenges of Eliminating or Substituting Antimicrobial Preservatives in Foods. Annu. Rev. Food Sci. Technol..

[13] Debonne E., Van Bockstaele F., Samapundo S., Eeckhout M., Devlieghere F. (2018). The use of essential oils as natural antifungal preservatives in bread products. J. Essent. Oil Res..

[14] Calo J.R., Crandall P.G., O’Bryan C.A., Ricke S.C. (2015). Essential oils as antimicrobials in food systems—A review. Food Control.

[15] Perricone M., Arace E., Corbo M.R., Sinigaglia M., Bevilacqua A. (2015). Bioactivity of essential oils: A review on their interaction with food components. Front. Microbiol..

[16] da Silva B.D., Bernardes P.C., Pinheiro P.F., Fantuzzi E., Roberto C.D. (2021). Chemical composition, extraction sources and action mechanisms of essential oils: Natural preservative and limitations of use in meat products. Meat Sci..

[17] Ultee A., Bennik M.H., Moezelaar R. (2002). The phenolic hydroxyl group of carvacrol is essential for action against the food-borne pathogen *Bacillus cereus*. Appl. Environ. Microbiol..

[18] Veldhuizen E.J., Tjeerdsma-van Bokhoven J.L., Zweijtzer C., Burt S.A., Haagsman H.P. (2006). Structural requirements for the antimicrobial activity of carvacrol. J. Agric. Food Chem..

[19] Dufour V., Stahl M., Rosenfeld E., Stintzi A., Baysse C. (2013). Insights into the mode of action of benzyl isothiocyanate on *Campylobacter jejuni*. Appl. Environ. Microbiol..

[20] Lv F., Liang H., Yuan Q., Li C. (2011). In vitro antimicrobial effects and mechanism of action of selected plant essential oil combinations against four food-related microorganisms. Food Res. Int..

[21] Caesar L.K., Cech N.B. (2019). Synergy and antagonism in natural product extracts: When 1 + 1 does not equal 2. Nat. Prod. Rep..

[22] Debonne E., Vermeulen A., Van Bockstaele F., Soljic I., Eeckhout M., Devlieghere F. (2019). Growth/no-growth models of in-vitro growth of *Penicillium paneum* as a function of thyme essential oil, pH, a(w), temperature. Food Microbiol..

[23] Debonne E., Van Bockstaele F., De Leyn I., Devlieghere F., Eeckhout M. (2018). Validation of in-vitro antifungal activity of thyme essential oil on *Aspergillus niger* and *Penicillium paneum* through application in par-baked wheat and sourdough bread. LWT.

[24] Burt S. (2004). Essential oils: Their antibacterial properties and potential applications in foods—A review. Int. J. Food Microbiol..

[25] Salim-ur-Rehman, Hussain S., Nawaz H., Ahmad M.M., Murtaza M.A., Rizvi A.J. (2007). Inhibitory Effect of Citrus Peel Essential Oils on the Microbial Growth of Bread. Pak. J. Nutr..

[26] Fadiji T., Rashvand M., Daramola M.O., Iwarere S.A. (2023). A Review on Antimicrobial Packaging for Extending the Shelf Life of Food. Processes.

[27] Feyaerts A.F., Mathé L., Luyten W., De Graeve S., Van Dyck K., Broekx L., Van Dijck P. (2018). Essential oils and their components are a class of antifungals with potent vapour-phase-mediated anti-*Candida* activity. Sci. Rep..

[28] (2004). Method for Antifungal Disk Diffusion Susceptibility Testing of Yeast (M44-A).

[29] Abramoff M.D., Magalhaes P.J., Ram S.J. (2004). Image Processing with ImageJ. Biophotonics Int..

[30] (2008). EUCAST Technical Note on the method for the determination of broth dilution minimum inhibitory concentrations of antifungal agents for conidia-forming moulds. Clin. Microbiol. Infect..

[31] Bellio P., Fagnani L., Nazzicone L., Celenza G. (2021). New and simplified method for drug combination studies by checkerboard assay. MethodsX.

[32] Meyer C.T., Wooten D.J., Paudel B.B., Bauer J., Hardeman K.N., Westover D., Lovly C.M., Harris L.A., Tyson D.R., Quaranta V. (2019). Quantifying Drug Combination Synergy along Potency and Efficacy Axes. Cell Syst..

[33] Fisher K., Phillips C. (2008). Potential antimicrobial uses of essential oils in food: Is citrus the answer?. Trends Food Sci. Technol..

[34] Hossain T.J. (2024). Methods for screening and evaluation of antimicrobial activity: A review of protocols, advantages, and limitations. Eur. J. Microbiol. Immunol..

[35] Gandova V., Lazarov A., Fidan H., Dimov M., Stankov S., Denev P., Ercisli S., Stoyanova A., Gulen H., Assouguem A. (2023). Physicochemical and biological properties of carvacrol. Open Chem..

[36] Fahey J.W., Zalcmann A.T., Talalay P. (2001). The chemical diversity and distribution of glucosinolates and isothiocyanates among plants. Phytochemistry.

[37] Inouye S., Uchida K., Maruyama N., Yamaguchi H., Abe S. (2006). A novel method to estimate the contribution of the vapor activity of essential oils in agar diffusion assay. Nihon Ishinkin Gakkai Zasshi.

[38] Bhattacharyya A., Sinha M., Singh H., Patel R.S., Ghosh S., Sardana K., Ghosh S., Sengupta S. (2020). Mechanistic Insight into the Antifungal Effects of a Fatty Acid Derivative Against Drug-Resistant Fungal Infections. Front. Microbiol..

[39] Friedman M., Henika P.R., Mandrell R.E. (2002). Bactericidal activities of plant essential oils and some of their isolated constituents against *Campylobacter jejuni*, *Escherichia coli*, *Listeria monocytogenes*, and *Salmonella enterica*. J. Food Prot..

[40] Schlösser I., Prange A. (2018). Antifungal activity of selected natural preservatives against the foodborne molds *Penicillium verrucosum* and *Aspergillus westerdijkiae*. FEMS Microbiol. Lett..

[41] Pei R.S., Zhou F., Ji B.P., Xu J. (2009). Evaluation of combined antibacterial effects of eugenol, cinnamaldehyde, thymol, and carvacrol against *E. coli* with an improved method. J. Food Sci..

[42] Soković M., Glamočlija J., Marin P.D., Brkić D., van Griensven L.J. (2010). Antibacterial effects of the essential oils of commonly consumed medicinal herbs using an in vitro model. Molecules.

[43] Bassolé I.H., Lamien-Meda A., Bayala B., Tirogo S., Franz C., Novak J., Nebié R.C., Dicko M.H. (2010). Composition and antimicrobial activities of *Lippia multiflora* Moldenke, *Mentha × piperita* L. and *Ocimum basilicum* L. essential oils and their major monoterpene alcohols alone and in combination. Molecules.

[44] Wendakoon C.N., Sakaguchi M. (1993). Combined Effect of Sodium Chloride and Clove on Growth and Biogenic Amine Formation of *Enterobacter aerogenes* in Mackerel Muscle Extract. J. Food Protect..

[45] Donaldson J.R., Warner S.L., Cates R.G., Gary Young D. (2005). Assessment of Antimicrobial Activity of Fourteen Essential Oils When Using Dilution and Diffusion Methods. Pharm. Biol..

[46] Klancnik A., Piskernik S., Jersek B., Mozina S.S. (2010). Evaluation of diffusion and dilution methods to determine the antibacterial activity of plant extracts. J. Microbiol. Methods.

[47] Nielsen P.V., Rios R. (2000). Inhibition of fungal growth on bread by volatile components from spices and herbs, and the possible application in active packaging, with special emphasis on mustard essential oil. Int. J. Food Microbiol..

[48] Jang M., Hong E., Kim G.H. (2010). Evaluation of antibacterial activity of 3-butenyl, 4-pentenyl, 2-phenylethyl, and benzyl isothiocyanate in *Brassica* vegetables. J. Food Sci..

[49] Li P., Zhao Y.M., Wang C., Zhu H.P. (2021). Antibacterial activity and main action pathway of benzyl isothiocyanate extracted from papaya seeds. J. Food Sci..

[50] Clemente I., Aznar M., Salafranca J., Nerín C. (2017). Raman spectroscopy, electronic microscopy and SPME-GC-MS to elucidate the mode of action of a new antimicrobial food packaging material. Anal. Bioanal. Chem..

[51] Friedman M. (2014). Chemistry and multibeneficial bioactivities of carvacrol (4-isopropyl-2-methylphenol), a component of essential oils produced by aromatic plants and spices. J. Agric. Food Chem..

[52] Wijesundara N.M., Lee S.F., Cheng Z., Davidson R., Langelaan D.N., Rupasinghe H.P.V. (2022). Bactericidal Activity of Carvacrol against *Streptococcus pyogenes* Involves Alteration of Membrane Fluidity and Integrity through Interaction with Membrane Phospholipids. Pharmaceutics.

[53] Gill A.O., Holley R.A. (2004). Mechanisms of bactericidal action of cinnamaldehyde against *Listeria monocytogenes* and of eugenol against *L. monocytogenes* and *Lactobacillus sakei*. Appl. Environ. Microbiol..

[54] Shi C., Knøchel S. (2021). Susceptibility of dairy associated molds towards microbial metabolites with focus on the response to diacetyl. Food Control.

[55] Avis T.J., Bélanger R.R. (2001). Specificity and mode of action of the antifungal fatty acid cis-9-heptadecenoic acid produced by *Pseudozyma flocculosa*. Appl. Environ. Microbiol..

[56] Cava-Roda R.M., Taboada-Rodríguez A., Valverde-Franco M.T., Marín-Iniesta F. (2012). Antimicrobial Activity of Vanillin and Mixtures with Cinnamon and Clove Essential Oils in Controlling *Listeria monocytogenes* and *Escherichia coli* O157:H7 in Milk. Food Bioprocess. Technol..

[57] Firouzi R., Shekarforoush S.S., Nazer A.H., Borumand Z., Jooyandeh A.R. (2007). Effects of essential oils of oregano and nutmeg on growth and survival of *Yersinia enterocolitica* and *Listeria monocytogenes* in barbecued chicken. J. Food Prot..

[58] Gill A.O., Delaquis P., Russo P., Holley R.A. (2002). Evaluation of antilisterial action of cilantro oil on vacuum packed ham. Int. J. Food Microbiol..

[59] Guynot M.E., Ramos A.J., Setó L., Purroy P., Sanchis V., Marín S. (2003). Antifungal activity of volatile compounds generated by essential oils against fungi commonly causing deterioration of bakery products. J. Appl. Microbiol..

[60] Smith J.P., Daifas D.P., El-Khoury W., Koukoutsis J., El-Khoury A. (2004). Shelf life and safety concerns of bakery products—Areview. Crit. Rev. Food Sci. Nutr..

[61] Gutierrez J., Barry-Ryan C., Bourke P. (2008). The antimicrobial efficacy of plant essential oil combinations and interactions with food ingredients. Int. J. Food Microbiol..

[62] Weisany W., Yousefi S., Tahir N.A., Golestanehzadeh N., McClements D.J., Adhikari B., Ghasemlou M. (2022). Targeted delivery and controlled released of essential oils using nanoencapsulation: A review. Adv. Colloid Interface Sci..

[63] Pinilla C.M.B., Thys R.C.S., Brandelli A. (2019). Antifungal properties of phosphatidylcholine-oleic acid liposomes encapsulating garlic against environmental fungal in wheat bread. Int. J. Food Microbiol..

[64] Gonçalves N.D., Pena F.d.L., Sartoratto A., Derlamelina C., Duarte M.C.T., Antunes A.E.C., Prata A.S. (2017). Encapsulated thyme (*Thymus vulgaris*) essential oil used as a natural preservative in bakery product. Food Res. Int..

[65] Teodoro R.A.R., de Barros Fernandes R.V., Botrel D.A., Borges S.V., de Souza A.U. (2014). Characterization of Microencapsulated Rosemary Essential Oil and Its Antimicrobial Effect on Fresh Dough. Food Bioprocess. Technol..

