# Supplementary material for: The Potential of Plant Secondary Metabolites as Bread Mould Inhibitors: Exploring Their Individual and Combined Antifungal Effect
Source: Foods. 2025 Oct 23;14(21):3604. doi: 10.3390/foods14213604 (PMC12607509; doi:10.3390/foods14213604)
Supplement: Supplementary file 1 [file foods-14-03604-s001.zip › foods-3878778-supplementary.pdf]

Table S1: List of all plant secondary metabolites tested in this research, and their supplier.

	Compound	Supplier
1	Octanoic acid	Sigma Aldrich
2	Furfural	VWR
3	E-cinnamaldehyde	Ambeed
4	2,3-butanedione	Sigma Aldrich
5	Allylthiol	Sigma Aldrich
6	Allyl isothiocyanate	Sigma Aldrich
7	Carvacrol	Ambeed
8	3-Decenone-2	Ambeed
9	Hexanoic acid	Sigma Aldrich
10	Acetaldehyde	Sigma Aldrich
11	Butanal	Sigma Aldrich
12	alpha-Methylcinnamaldehyde	Sigma Aldrich
13	Benzyl isothiocyanate	Sigma Aldrich
14	9-Decen-1-ol	Ambeed
15	Anisaldehyde	Ambeed
16	Benzaldehyde	Sigma Aldrich
17	Cuminaldehyde	Ambeed
18	Citral	Ambeed
19	Cis-Jasmone	Ambeed
20	Cinnamyl alcohol	Ambeed
21	2,4-Dimethylbenzaldehyde	Ambeed
22	Cinnamyl acetate	Ambeed
23	2-Methoxy-4-propylphenol	Ambeed
24	Cumin alcohol	Ambeed
25	Dimethyl trisulfide	Sigma Aldrich
26	Benzyl thiol	Sigma Aldrich
27	(z)-butylidene phthalide	Ambeed
28	Creosol	Ambeed
29	Eugenol	Ambeed
30	4-Methyl-2,6-dimethoxyphenol	Sigma Aldrich
31	Nerol	Ambeed
32	Citronellal	Ambeed
33	Geraniol	Ambeed
34	1,2-Dimethoxybenzene	Ambeed
35	(-)-Carvone	Ambeed
36	Delta-dodecalactone	Ambeed
37	Anisyl acetate	Ambeed
38	Citronellol	Ambeed
39	Butanoic acid	Sigma Aldrich
40	Cinnamyl formate	Ambeed
41	3,4-Dihydrocoumarin	Ambeed
42	1-Decanol	Ambeed
43	(+)-Carvone	Ambeed
44	Dimethyl disulfide	Sigma Aldrich
45	Decanal	Ambeed
46	2,4-Dimethylacetophenone	Sigma Aldrich
47	Benzyl formate	Ambeed

48	Benzyl acetone	Ambeed
49	Delta-decalactone	Ambeed
50	Benzothiazole	Ambeed
51	Methyl n-methylanthranilate	Ambeed
52	Vanillin	Sigma Aldrich
53	2,5-Dimethylthiophene	Ambeed
54	delta-nonalactone	Ambeed
55	Benzyl phenylacetate	Sigma Aldrich
56	Anethole	Ambeed
57	Benzyl propionate	Ambeed
58	Trans-anethole	Ambeed
59	1,1-diethoxyethane	Sigma Aldrich
60	3-Methyloctano-1,4-lactone	Sigma Aldrich
61	Hydroxy tyrosol	Ambeed
62	2-Butylfuran	Ambeed
63	α-terpineol	Sigma Aldrich
64	Cinnamyl butyrate	Ambeed
65	Benzyl acetate	Ambeed
66	alpha-damascone	Ambeed
67	2-Acetyl-3-ethylpyrazine	Sigma Aldrich
68	Butyl phenylacetate	Sigma Aldrich
69	Benzyl methyl ether	Ambeed
70	delta-octalactone	Ambeed
71	Dihydromycrenol	Sigma Aldrich
72	Farnesol	Ambeed
73	Dihydrocarvone	Sigma Aldrich
74	2-Acetyl-5-methylfuran	Ambeed
75	Linalool	Ambeed
76	Benzyl alcohol	Ambeed
77	Butyl benzoate	Ambeed
78	Formic acid	Sigma Aldrich
79	1-Isopropenyl-4-methylbenzene	Sigma Aldrich
80	Anisole	Sigma Aldrich
81	(-)-Limonene	Ambeed
82	Dodecanal	Ambeed
83	Rutaecarpine	Ambeed
84	2,4-Dithiapentane	Sigma Aldrich
85	Butyl valerate	Ambeed
86	Dihydro-beta-ionone	Sigma Aldrich
87	Diallyl sulfide	Sigma Aldrich
88	2-Decanone	Ambeed
89	Benzyl isobutyrate	Ambeed
90	Cinnamyl cinnamate	Ambeed
91	Diethyl succinate	Ambeed
92	Benzyl cinnamate	Ambeed
93	2-Acetyl-3-methylpyrazine	Sigma Aldrich
94	d-Fenchone	Ambeed
95	Diethyl malonate	Sigma Aldrich
96	Benzyl benzoate	Sigma Aldrich
97	Acetic acid	Sigma Aldrich
98	Benzyl salicylate	Ambeed
99	beta-damascone	Sigma Aldrich

100	Butyl hexanoate	Ambeed
101	Octadecanoic acid	Ambeed
102	Piperine	Ambeed
103	Allo-ocimene	Sigma Aldrich
104	Cinnamyl isovalerate	Ambeed
105	alpha-ionol	Sigma Aldrich
106	2-Acetylthiazole	Ambeed
107	Anisalcohol	Ambeed
108	Furfuryl alcohol	Sigma Aldrich
109	Menthol	Ambeed
110	Diethyl malate	Ambeed
111	3-Decanone	Sigma Aldrich
112	Diethyl disulfide	Sigma Aldrich
113	Nerolidol	Ambeed
114	Butyl isovalerate	Ambeed
115	Dodecan-1-ol	Sigma Aldrich
116	Sinapate	Ambeed
117	Butyl 2-methylbutyrate	Ambeed
118	Butyl butyrate	Sigma Aldrich
119	(9Z)-Octadecenoic acid	Ambeed
120	Naringin	Ambeed
121	Butyl isobutyrate	Ambeed
122	Farnesyl acetate	Sigma Aldrich
123	Diethyl carbonate	Sigma Aldrich
124	Citronellyl formate	Ambeed
125	Methyl salicylate	Ambeed
126	Dodecanoic acid	Ambeed
127	2-acetylfuran	Ambeed
128	Hexadecanoic acid	Sigma Aldrich
129	Myrcene	Sigma Aldrich
130	Caffeine	Sigma Aldrich
131	Diethyl oxalate	Sigma Aldrich
132	Geranyl acetate	Ambeed
133	Butyl acetate	Sigma Aldrich
134	Cyclohexanol	Sigma Aldrich
135	D3-carene	Sigma Aldrich
136	Dimethyl malonate	Ambeed
137	Hesperetin	Ambeed
138	1,4-Cineole	Sigma Aldrich
139	Dipropyl disulfide	Ambeed
140	Tyramine	Sigma Aldrich
141	α-terpinene	Ambeed
142	Theobromine	Sigma Aldrich
143	5-(2-Hydroxyethyl)-4-methylthiazole	Ambeed
144	2,5-Dimethyl-4-methoxyfuran-3(2H)-one	Sigma Aldrich
145	Benzophenone	Ambeed
146	Benzyl butyrate	Ambeed
147	Decanoic acid	Ambeed
148	Diethyl sulfide	Sigma Aldrich
149	alpha-Hexylcinnamaldehyde	Ambeed
150	Citronellyl acetate	Ambeed
151	Linoleate	Ambeed

152	Myrtenyl acetate	Sigma Aldrich
153	α-Phellandrene	Ambeed
154	beta-Caryophyllene epoxide	Sigma Aldrich
155	2-Phenylpropan-2-ol	Ambeed
156	1,1-Diethoxyhexane	Ambeed
157	2-Acetylpyridine	Ambeed
158	Cyclopentanone	Sigma Aldrich
159	2,3-Dimethylpyrazine	Ambeed
160	Citronellyl isobutyrate	Sigma Aldrich
161	2-Acetylpyrrole	Ambeed
162	Diphenyl oxide	Ambeed
163	2-Butanol	Sigma Aldrich
164	alpha-picoline	Sigma Aldrich
165	1,8-Cineole	Sigma Aldrich
166	Thymol	Sigma Aldrich
167	Isoamyl 2-methylbutyrate	Sigma Aldrich
168	Benzyl isovalerate	Ambeed
169	2,6-Dimethylpyrazine	Ambeed
170	Cinnamic acid	Ambeed
171	Neryl acetate	Ambeed
172	Fenchyl acetate	Sigma Aldrich
173	Citronellyl tiglate	Sigma Aldrich
174	Linalyl acetate	Ambeed
175	Cyclohexanone	Sigma Aldrich
176	γ-Terpinene	Sigma Aldrich
177	Azelaic acid	Ambeed
178	1,1-Dimethoxyethane	Sigma Aldrich
179	Cuminal	Ambeed
180	2,5-Dimethylfuran	Sigma Aldrich
181	Dibenzyl disulfide	Ambeed
182	Butyl propionate	Sigma Aldrich
183	Tetradecanoic acid	Ambeed
184	Ethyl acetate	Ambeed
185	Citronellyl propionate	Ambeed
186	Phloretin	Sigma Aldrich
187	p-Cymene	Sigma Aldrich
188	(-)-Bornyl acetate	Ambeed
189	Cedryl acetate	Ambeed
190	Dibutyl disulfide	Sigma Aldrich
191	Dimethyl sulfide	Sigma Aldrich
192	2,6-Dimethylphenol	Sigma Aldrich
193	β-Caryophyllene	Ambeed
194	1,4-Dimethoxybenzene	Ambeed
195	3,5-Dimethylphenol	Sigma Aldrich
196	Veratraldehyde	Ambeed
197	o-methoxycinnamaldehyde	Ambeed
198	Dihydrocarvyl acetate	Sigma Aldrich
199	Cedrol	Ambeed
200	Anisic acid	Ambeed
201	Dodecane	Ambeed
202	2,5-Dimethylpyrazine	Ambeed
203	Acetovanillone	Ambeed

204	2,5-Dimethylphenol	Sigma Aldrich
205	2,5-Dimethylfuran-3(2H)-one	Sigma Aldrich
206	Camphene	Sigma Aldrich
207	Decyl acetate	Ambeed
208	delta-hexalactone	Ambeed
209	Camphor	Ambeed
210	2-Butanone	Sigma Aldrich
211	Butane-1,3-diol	Ambeed
212	1-Butanol	Sigma Aldrich
213	Menthone	Sigma Aldrich
214	Terpinyl acetate	Sigma Aldrich
215	Ambrettolide	Sigma Aldrich
216	Acetone	VWR
217	Benzoic acid	Sigma Aldrich
218	Furaneol	Ambeed

Table S2: Antifungal activity of essential oil compounds and plant secondary compounds against ten moulds isolated from spoiled par-baked bread. (A) zone of inhibition in mm, obtained by performing the agar disk diffusion assay with 1% compound solution, and (B) relative growth inhibition (%), in broth dilution assay with 0.05% compound solution.

		P. bialowiezense		*P. brevicompactum*		*P. crustosum*		*P. palitans*		*P. hordei*		*P. polonicum*		*P. chrysogenum*		*P. corylophilum*		*P. glabrum*		*A. westerdijkiae*	
		A	B	A	B	A	B	A	B	A	B	A	B	A	B	A	B	A	B	A	B
1	Octanoic acid	11	100	8	95.6	8	100	8	100	12	98.9	7	100	12	100	8	99.8	8	100	7	100
2	Furfural	6	100	9	100	10	99.3	8	98.2	8	98.6	7	100	10	100	6	98.4	6	100	6	99.5
3	E-cinnamaldehyde	14	100	15	100	13	100	14	100	8	100	16	100	8	97.5	11	96.5	6	100	15	99.8
4	2,3-butanedione	7	100	8	100	10	91.5	6	97.5	6	99.2	6	100	7	100	7	96.7	6	99.5	8	99.3
5	Allylthiol	6	100	10	100	7	100	7	100	7	93.9	8	94.0	7	100	6	96.5	6	100	6	98.5
6	Allyl isothiocyanate	12	100	6	91.2	6	91.8	6	94.1	6	100	6	100	8	100	6	100	6	100	11	99.9
7	Carvacrol	11	100	11	95.0	11	89.1	9	95.8	8	98.8	9	100	12	100	8	97.1	11	98.7	9	99.4
8	3-Decenone-2	6	100	10	100	9	91.2	11	92.8	6	99.6	10	92.4	9	100	6	96.5	6	99.7	6	99.9
9	Hexanoic acid	8	100	8	100	10	80.3	9	100	10	95.0	7	100	7	98.5	8	96.0	6	99.5	7	96.2
10	Acetaldehyde	6	100	9	91.2	9	100	6	100	6	100	6	79.0	7	98.4	6	94.2	6	98.4	6	100
11	Butanal	6	100	8	100	9	81.3	10	81.3	6	100	9	100	7	99.3	6	91.1	6	100	6	100
12	alpha-Methylcinnamaldehyde	6	100	9	100	7	86.8	9	100	6	99.1	8	68.9	7	98.2	6	93.6	6	100	6	99.7
13	Benzyl isothiocyanate	51	100	90	100	90	88.5	90	79.5	27	100	90	78.6	13	100	90	100	36	98.4	46	99.7
14	9-Decen-1-ol	6	100	12	96.9	10	100	8	100	7	98.6	7	83.5	7	94.9	8	92.3	6	77.4	9	100
15	Anisaldehyde	6	100	6	87.6	9	100	6	100	6	99.1	6	57.1	7	98.0	6	93.8	6	95.8	6	98.8
16	Benzaldehyde	6	100	10	100	8	84.5	9	69.8	6	99.1	8	66.3	6	100	6	93.1	6	98.3	6	99.7
17	Cuminaldehyde	6	97.3	12	100	7	83.4	7	73.6	6	98.6	6	74.5	8	100	6	85.5	6	95.7	6	98.7

18	Citral	9	100	10	80.2	11	88.0	9	86.1	6	99.1	9	57.6	7	98.0	6	81.2	6	97.8	6	100
19	Cis-Jasmone	6	95.0	7	100	7	96.2	9	79.4	6	84.4	9	61.6	9	95.6	6	84.7	6	92.4	6	96.4
20	Cinnamyl alcohol	6	100	9	100	9	83.3	10	95.4	7	82.3	9	93.0	9	94.9	7	79.9	7	79.6	6	70.5
21	2,4-Dimethylbenzal-dehyde	6	91.8	7	100	9	85.6	9	100	6	97.7	9	14.0	6	99.4	6	87.9	6	95.8	9	96.4
22	Cinnamyl acetate	6	100	10	82.3	7	91.9	10	100	6	100	7	63.2	6	93.4	6	68.0	6	95.2	6	72.2
23	2-Methoxy-4-propylphenol	11	100	8	93.0	9	98.9	9	96.1	7	88.8	9	100	11	0.0	8	93.2	8	99.7	6	79.1
24	Cumin alcohol	6	99.4	7	97.6	8	83.6	8	87.5	6	97.3	8	29.9	13	97.4	7	63.9	8	93.8	6	98.7
25	Dimethyl trisulfide	6	100	10	100	10	100	10	100	7	100	10	47.0	7	0.0	6	97.7	6	100	6	99.6
26	Benzyl thiol	6	100	9	87.0	10	98.8	11	70.8	7	0.0	9	100	9	100	6	86.6	6	98.9	7	91.1
27	(z)-butylidene phthalide	6	100	11	88.5	12	91.8	6	97.3	7	49.2	7	74.2	6	94.5	6	75.2	6	95.1	9	66.0
28	Creosol	6	100	10	93.5	7	100	9	90.9	7	99.5	9	60.5	9	0.0	8	93.6	6	98.2	6	95.3
29	Eugenol	9	100	8	97.0	9	59.9	8	96.1	6	99.0	8	84.7	7	0.0	7	94.9	7	100	8	99.5
30	4-Methyl-2,6-di-methoxyphenol	6	99.2	9	89.7	8	90.9	9	91.1	6	83.4	9	13.4	8	92.8	7	68.6	6	81.7	8	82.9
31	Nerol	6	98.0	8	71.1	8	90.2	9	100	6	77.5	8	42.6	10	85.6	6	57.8	8	81.4	6	86.9
32	Citronellal	7	87.3	10	75.4	10	79.9	10	100	8	96.4	9	46.7	10	95.8	6	13.8	6	93.3	9	92.8
33	Geraniol	6	95.0	7	94.5	8	90.3	9	94.5	6	76.6	8	78.3	11	84.3	7	0.0	10	82.1	6	83.9
34	1,2-Dimethoxyben-zene	6	92.2	6	79.6	9	93.4	10	85.6	6	64.7	10	12.1	7	87.3	6	77.6	6	85.9	6	86.9
35	(-)-Carvone	6	100	12	91.1	8	82.8	13	87.8	6	97.2	12	21.7	8	95.5	6	23.8	6	89.9	6	59.6
36	Delta-dodecalactone	7	64.0	11	100	11	89.8	10	100	8	13.2	10	69.5	11	100	8	89.6	8	95.5	6	25.2
37	Anisyl acetate	6	99.7	6	93.7	6	84.3	9	78.1	6	94.6	9	0.0	6	94.5	6	11.9	6	90.4	6	94.8
38	Citronellol	6	92.9	9	100	11	84.6	12	85.1	7	38.8	11	79.9	16	80.4	7	0.0	6	51.3	8	79.7

39	Butanoic acid	6	95.7	7	95.7	7	82.0	9	64.0	7	56.0	9	68.5	6	88.3	7	17.2	8	87.2	6	36.3
40	Cinnamyl formate	6	100	7	89.0	11	91.3	11	81.3	6	0.0	10	59.6	7	70.8	6	32.8	6	74.5	6	86.1
41	3,4-Dihydrocouma-rin	6	75.5	9	80.6	10	75.4	8	75.0	10	100	8	0.0	15	98.5	6	0.0	13	100	6	76.0
42	1-Decanol	6	83.8	11	98.2	10	75.1	10	90.6	7	51.7	9	88.4	7	57.8	7	23.4	12	42.6	7	47.8
43	(+)-Carvone	7	100	9	75.6	11	92.1	10	77.2	6	86.2	9	28.6	8	93.2	6	18.8	6	4.3	6	78.0
44	Dimethyl disulfide	6	77.0	7	86.4	8	48.1	9	9.9	6	70.3	9	74.2	7	77.0	7	44.8	6	65.5	7	96.7
45	Decanal	7	72.2	10	100	9	66.5	9	43.8	7	29.9	9	67.0	7	99.5	6	18.3	6	85.5	8	45.6
46	2,4-Dimethylaceto-phenone	6	100	9	87.3	10	64.1	10	60.0	6	43.6	9	50.1	9	66.1	6	52.4	6	88.4	7	0.0
47	Benzyl formate	6	84.5	7	77.9	9	79.9	9	57.7	6	72.3	8	32.6	9	45.5	7	57.3	6	51.3	6	44.0
48	Benzyl acetone	6	84.2	10	94.7	10	79.6	9	79.7	6	49.6	9	27.3	7	0.0	6	70.1	6	43.0	9	65.4
49	Delta-decalactone	7	99.7	10	100	11	82.7	10	97.0	7	0.0	9	37.2	9	44.0	7	28.7	6	79.8	8	20.5
50	Benzothiazole	6	85.0	10	94.4	9	89.5	8	44.4	7	51.3	8	0.5	10	100	6	9.3	6	96.4	7	13.7
51	Methyl n-methylan-thranilate	6	99.4	8	64.4	8	55.4	10	92.9	6	67.2	10	25.3	6	0.0	6	70.3	6	87.3	6	16.7
52	Vanillin	6	38.6	6	81.8	7	58.2	6	63.9	6	6.4	7	92.6	8	89.3	7	36.9	6	100	8	0.0
53	2,5-Dimethylthio-phene	6	98.0	10	80.8	8	100	6	66.1	6	2.5	7	0.0	7	100	7	12.1	6	80.8	6	9.2
54	delta-nonalactone	6	80.4	9	100	9	91.1	9	46.2	8	1.2	9	59.2	7	67.9	8	9.8	6	76.5	6	15.5
55	Benzyl phenylacetate	7	88.8	9	66.0	10	78.3	10	63.8	6	20.0	10	15.4	9	59.3	6	71.6	6	57.6	6	16.8
56	Anethole	6	86.4	9	58.5	13	56.7	8	46.8	6	54.9	8	31.5	6	62.7	6	47.1	6	88.1	6	0.0
57	Benzyl propionate	6	88.6	9	64.1	9	27.2	8	74.7	6	48.9	8	23.4	9	53.4	6	0.0	6	84.9	6	64.2
58	Trans-anethole	6	79.3	8	71.5	9	74.5	8	68.9	6	41.7	8	0.0	6	29.9	6	57.5	6	88.2	7	5.3
59	1,1-diethoxyethane	7	79.7	8	100	7	50.6	8	25.9	7	0.0	7	79.1	7	94.1	8	0.0	7	82.3	6	0.0

60	3-Methyloctano-1,4-lactone	6	100	9	80.0	10	54.7	6	83.3	7	45.7	7	5.1	8	61.4	7	0.0	6	39.9	6	34.5
61	Hydroxy tyrosol	7	37.9	6	79.1	7	65.9	6	67.8	6	31.0	8	89.9	8	0.0	7	41.4	6	86.3	6	0.0
62	2-Butylfuran	6	51.5	9	54.5	9	69.8	8	18.4	6	0.0	8	34.0	11	66.9	7	62.6	6	65.1	6	74.6
63	α-terpineol	7	69.8	9	28.3	9	0.0	8	65.3	6	32.6	8	29.9	11	79.8	7	37.0	8	62.8	8	62.7
64	Cinnamyl butyrate	6	69.2	9	54.3	6	74.6	9	67.1	6	22.1	8	37.0	6	65.2	6	0.0	6	78.3	6	0.0
65	Benzyl acetate	6	71.8	9	95.2	9	41.1	9	42.1	6	45.0	9	84.2	8	0.0	8	19.4	6	34.9	6	22.7
66	alpha-damascone	7	73.0	7	64.6	9	67.6	10	43.8	6	55.8	11	16.1	6	64.0	6	36.8	6	0.0	8	34.4
67	2-Acetyl-3-ethylpyrazine	7	95.7	10	83.2	9	100	6	43.0	6	0.0	6	0.0	9	0.0	6	29.5	6	84.8	6	17.1
68	Butyl phenylacetate	7	58.6		80.3	8	64.0	7	18.1	6	28.1	7	37.6	6	69.7	6	0.0	6	78.9	6	9.0
69	Benzyl methyl ether	6	75.0	10	36.2	9	0.0	10	20.5	6	61.7	11	27.8	6	0.0	7	63.6	6	45.3	6	87.6
70	delta-octalactone	6	57.0	8	76.2	7	58.6	6	65.3	8	20.4	7	31.7	7	0.0	6	31.5	6	53.9	6	22.8
71	Dihydromycrenol	7	81.9	7	40.0	9	76.5	8	58.3	6	37.8	7	16.4	10	36.4	7	24.0	8	21.3	6	23.5
72	Farnesol	7	86.4	7	38.7	10	83.1	9	54.8	6	0.0	9	10.7	12	0.0	6	31.8	6	70.7	7	34.8
73	Dihydrocarvone	7	95.9	8	61.5	10	45.4	9	62.4	7	40.1	9	0.0	7	48.6	8	0.0	6	29.3	7	27.0
74	2-Acetyl-5-methylfuran	6	54.4	9	81.7	8	86.0	7	40.2	6	25.6	8	16.1	9	6.9	7	34.9	6	27.8	6	27.4
75	Linalool	6	0.4	8	32.7	8	83.8	9	79.8	6	41.1	9	43.9	10	1.8	7	30.1	7	34.4	6	49.7
76	Benzyl alcohol	7	77.4	7	57.7	9	59.7	10	51.6	7	14.4	9	75.0	8	0.0	7	6.3	8	25.0	6	16.1
77	Butyl benzoate	6	72.9	10	23.1	8	58.2	6	46.3	6	14.0	6	40.7	8	0.0	6	58.7	6	57.6	6	0.0
78	Formic acid	6	92.9	9	32.7	8	42.6	8	49.3	7	47.3	8	46.5	9	27.4	6	0.0	6	0.0	6	30.3
79	1-Isopropenyl-4-methylbenzene	7	73.9		78.1	6	34.8	10	80.6	7	46.2	9	7.8	8	38.3	9	0.0	6	9.0	6	0.0
80	Anisole	6	53.8	7	74.5	8	39.0	10	21.8	6	50.6	10	19.4	9	2.9	7	4.2	6	83.5	9	2.3

81	(-)-Limonene	7	75.7	9	62.5	8	51.0	9	67.5	6	0.0	9	0.0	7	30.6	6	0.0	6	62.5	6	0.0
82	Dodecanal	6	60.7	12	24.9	11	71.2	11	6.3	7	0.0	10	4.8	6	43.9	7	0.0	6	82.4	8	52.6
83	Rutaecarpine	6	64.3	8	0.0	6	65.9	8	25.8	6	19.6	7	95.0	6	15.2	8	14.1	6	28.2	7	9.4
84	2,4-Dithiapentane	7	57.7	10	90.9	8	69.5	11	54.8	6	0.0	9	56.4	7	6.4	7	0.0	7	0.0	6	1.8
85	Butyl valerate	6	55.6	6	4.2	7	30.8	6	49.1	6	13.6	7	35.9	9	0.0	7	61.2	6	77.4	8	4.3
86	Dihydro-beta-ionone	6	79.2	8	0.0	11	45.2	6	78.4	6	14.4	6	40.8	6	18.7	6	0.0	6	11.8	6	40.8
87	Diallyl sulfide	6	63.7	11	51.2	9	49.3	11	35.8	6	22.3	11	58.2	8	0.0	7	10.2	6	33.1	6	0.0
88	2-Decanone	7	40.4	9	52.8	10	42.3	7	16.6	6	36.4	7	33.4	9	16.0	7	15.8	6	65.5	6	0.0
89	Benzyl isobutyrate	6	79.6	9	19.5	9	22.7	9	63.3	6	28.9	8	23.1	7	16.8	6	15.2	6	47.7	6	0.0
90	Cinnamyl cinnamate	6	54.0	8	0.0	8	26.2	8	55.2	6	6.8	8	18.3	8	10.1	6	56.3	6	72.7	6	11.9
91	Diethyl succinate	6	58.1	7	74.2	9	16.7	9	19.3	6	33.8	9	31.4	8	8.6	7	0.0	6	47.9	6	12.2
92	Benzyl cinnamate	6	74.9	9	0.0	9	6.9	8	46.8	6	13.2	7	19.4	9	0.0	7	61.5	6	69.3	6	9.3
93	2-Acetyl-3-methylpy-razine	6	91.4	8	35.0	10	86.5	7	0.0	6	0.0	8	14.9	7	0.0	7	25.5	6	25.2	6	22.2
94	d-Fenchone	6	53.2	10	5.9	8	65.5	7	0.0	6	36.4	8	39.6	8	0.6	7	22.5	6	19.9	7	54.3
95	Diethyl malonate	6	71.5	8	47.6	8	56.6	9	52.8	6	0.0	8	52.6	7	0.0	8	0.0	7	4.0	6	12.0
96	Benzyl benzoate	6	75.7	6	38.9	9	11.3	6	54.4	6	0.0	7	37.7	7	0.0	7	3.6	6	5.0	6	68.7
97	Acetic acid	7	44.7	8	8.7	8	74.1	8	32.1	6	19.6	8	44.2	7	3.9	8	13.3	7	40.0	6	14.5
98	Benzyl salicylate	6	70.6	7	0.0	8	32.7	8	21.8	6	0.0	8	22.7	8	0.0	7	58.4	6	68.3	6	19.2
99	beta-damascone	6	56.1	11	0.0	8	0.0	6	31.8	6	33.6	6	44.3	7	16.4	6	23.9	6	19.9	6	67.0
100	Butyl hexanoate	6	73.7	9	0.0	7	5.7	6	63.0	6	4.7	6	34.1	8	0.0	7	49.4	6	59.3	6	0.0
101	Octadecanoic acid	6	59.1	7	15.5	8	60.8	8	0.0	6	3.2	7	89.3	9	14.2	7	15.1	6	30.7	7	0.0
102	Piperine	7	62.5	9	0.0	7	69.8	8	0.0	6	31.0	7	90.7	6	9.5	7	0.0	6	5.4	8	16.2

103	Allo-ocimene	7	54.4	9	22.4	8	0.0	6	66.8	6	0.0	6	0.0	8	25.8	8	58.7	6	41.8	7	10.3
104	Cinnamyl isovalerate	6	80.4	10	11.9	7	42.8	9	56.7	6	0.0	10	0.0	8	0.0	6	16.8	6	64.3	6	4.1
105	alpha-ionol	6	95.5	11	32.8	10	53.1	10	6.3	6	0.0	10	0.0	10	0.0	6	21.4	6	67.6	8	0.0
106	2-Acetylthiazole	6	95.1	9	28.7	9	37.5	6	38.3	6	0.0	6	0.0	7	0.0	6	12.6	6	63.4	6	0.0
107	Anisalcohol	6	50.9	7	0.0	8	55.2	9	53.4	6	0.0	8	73.8	8	18.5	7	0.0	7	10.3	6	12.0
108	Furfuryl alcohol	9	57.7	9	21.6	8	4.8	6	39.6	8	21.4	6	38.0	9	8.4	7	54.1	6	28.3	6	0.0
109	Menthol	6	0.0	9	7.0	9	57.7	8	0.0	6	6.8	7	28.9	10	42.9	6	34.6	7	68.2	6	24.8
110	Diethyl malate	6	83.7	8	0.0	7	25.7	8	50.4	6	15.7	8	7.0	8	0.0	8	64.5	6	10.0	7	7.7
111	3-Decanone	6	51.5	8	41.6	8	36.7	6	24.3	6	34.8	6	18.2	7	0.0	7	5.8	6	39.4	6	11.8
112	Diethyl disulfide	7	44.3	10	84.7	8	62.8	8	21.5	6	11.5	8	37.0	7	0.0	7	0.0	6	0.0	6	0.0
113	Nerolidol	7	77.0	11	19.4	10	68.1	9	31.4	6	0.0	9	0.0	9	0.0	7	0.0	6	61.6	8	0.0
114	Butyl isovalerate	6	29.4	8	50.2	8	11.9	9	6.9	6	24.2	9	70.6	7	0.0	7	0.0	6	63.2	6	0.0
115	Dodecan-1-ol	6	39.9	10	68.1	9	12.1	6	61.2	6	0.0	7	37.5	9	0.0	6	5.8	6	12.9	6	9.5
116	Sinapate	6	65.3	8	0.0	7	62.3	7	0.0	6	28.1	7	59.8	6	0.0	8	6.3	6	24.7	7	0.0
117	Butyl 2-methylbutyrate	6	50.9	9	23.8	17	10.8	7	15.7	6	28.3	7	47.1	7	0.0	7	5.4	6	38.6	6	25.7
118	Butyl butyrate	6	51.4	9	61.1	8	0.0	10	0.0	6	33.1	9	55.6	7	0.0	7	0.0	6	44.6	6	0.0
119	(9Z)-Octadecenoic acid	6	17.8	8	0.0	9	56.3	6	6.8	6	5.3	7	66.7	6	31.4	7	0.0	6	50.5	8	9.2
120	Naringin	8	55.8	7	24.9	6	62.6	6	0.0	6	27.0	7	61.0	6	0.9	8	0.0	6	9.7	9	1.5
121	Butyl isobutyrate	6	42.9	8	25.2	8	0.5	10	27.5	6	21.6	9	65.5	7	0.0	6	7.8	6	51.2	6	0.0
122	Farnesyl acetate	6	76.5	8	0.0	7	69.2	9	31.1	6	0.0	9	0.0	9	0.0	6	0.0	6	63.6	6	0.0
123	Diethyl carbonate	6	73.1	6	16.1	9	2.6	7	25.8	8	0.0	7	0.0	7	0.0	6	52.1	6	57.3	6	11.0
124	Citronellyl formate	6	56.7	9	0.0	9	28.3	9	59.2	6	6.4	8	0.0	8	0.0	7	53.9	6	0.1	6	32.8

125	Methyl salicylate	6	83.7	6	59.3	9	0.0	6	43.0	6	0.0	6	0.0	8	0.0	6	14.0	6	36.7	6	0.0
126	Dodecanoic acid	12	49.6	7	0.0	7	50.5	10	32.6	6	0.0	9	65.2	6	0.0	6	3.0	10	19.0	7	14.3
127	2-acetylfuran	6	0.0	9	27.6	9	63.2	7	0.0	6	13.6	7	38.1	9	0.0	8	26.8	6	37.5	6	27.1
128	Hexadecanoic acid	6	61.2	6	11.8	6	25.0	6	5.1	6	24.6	6	62.2	6	38.9	6	0.0	6	0.0	6	1.7
129	Myrcene	6	59.5	9	14.9	9	9.7	9	65.2	6	5.7	9	5.1	7	38.8	7	2.5	6	28.1	8	0.0
130	Caffeine	7	43.2	8	18.1	7	37.5	8	36.6	7	20.3	8	55.6	6	0.0	8	0.0	6	16.9	6	0.0
131	Diethyl oxalate	8	15.6	7	27.8	9	45.6	10	18.5	6	2.2	10	57.9	7	0.0	7	19.0	6	31.8	6	9.8
132	Geranyl acetate	6	48.8	11	23.8	11	0.0	8	41.6	6	11.9	8	12.4	6	25.1	6	6.9	6	39.7	10	16.5
133	Butyl acetate	6	48.1	9	58.0	6	0.0	7	38.0	6	29.4	7	26.6	7	1.4	7	4.2	6	17.8	7	2.1
134	Cyclohexanol	7	26.0	9	15.8	9	18.3	7	6.3	6	0.0	7	54.9	10	41.4	7	8.5	7	21.4	6	29.8
135	D3-carene	6	67.3	9	50.1	8	0.0	8	59.9	6	0.4	9	9.7	8	0.0	7	11.1	6	20.7	6	0.0
136	Dimethyl malonate	6	66.0	9	0.0	7	30.1	7	30.7	6	0.0	8	55.3	7	0.0	7	0.0	8	31.1	6	0.0
137	Hesperetin	6	38.7	7	8.4	7	57.2	6	6.8	6	23.1	7	44.6	8	18.0	7	0.0	6	16.3	7	0.0
138	1,4-Cineole	6	26.1	9	6.1	8	46.1	8	63.2	6	18.2	7	0.0	9	20.6	6	0.0	6	8.2	6	23.3
139	Dipropyl disulfide	7	0.0	9	47.5	8	25.6	7	32.2	6	21.2	7	70.6	7	0.0	8	11.2	6	0.0	6	1.0
140	Tyramine	9	11.8	8	16.2	8	64.3	8	39.3	6	11.4	7	39.2	6	6.4	7	2.0	6	16.5	6	0.0
141	α-terpinene	6	66.1	10	0.0	9	0.0	7	64.0	6	0.0	6	0.0	8	20.0	6	9.5	6	46.8	10	0.4
142	Theobromine	7	49.0	8	27.9	8	14.5	6	23.6	6	19.9	7	34.1	6	0.0	7	0.0	6	37.4	7	0.0
143	5-(2-Hydroxyethyl)-4-methylthiazole	7	34.7	9	0.0	7	14.2	8	1.8	6	8.2	7	91.8	8	23.2	8	0.0	6	19.4	7	12.8
144	2,5-Dimethyl-4-methoxyfuran-3(2H)-one	6	68.8	9	0.0	10	0.0	8	0.0	6	0.9	8	0.0	6	0.0	7	57.9	6	60.9	6	16.7
145	Benzophenone	6	32.9		6.1	7	0.0	9	17.7	6	22.6	9	1.0	9	43.6	6	2.4	6	64.4	6	12.1

146	Benzyl butyrate	6	92.3	8	6.1	8	40.0	7	12.9	6	10.6	7	16.5	7	0.0	6	0.0	6	24.1	6	0.0
147	Decanoic acid	22	51.4	7	0.0	9	39.3	10	55.5	7	0.0	10	11.7	11	9.3	6	2.2	6	31.4	12	0.0
148	Diethyl sulfide	7	65.9	8	41.5	9	25.6	6	0.0	6	0.0	6	0.0	8	0.0	7	0.0	6	54.5	6	12.1
149	alpha-Hexylcin-namaldehyde	8	20.8	9	5.1	9	20.3	11	11.1	6	0.0	10	44.1	7	16.3	6	11.2	6	61.8	6	6.8
150	Citronellyl acetate	6	40.0	7	0.0	9	57.5	9	0.0	6	20.1	10	21.1	9	0.0	6	7.8	6	19.9	6	28.8
151	Linoleic acid	9	0.0	7	0.0	7	59.4	8	0.0	9	4.6	7	47.6	6	0.0	7	18.9	6	59.0	6	3.8
152	Myrtenyl acetate	7	57.2	9	7.3	10	0.0	9	67.2	6	11.7	9	0.5	6	19.8	6	4.5	6	0.0	7	24.1
153	α-Phellandrene	7	59.9	9	0.0	8	30.3	6	66.8	6	1.6	7	23.0	7	0.0	6	7.0	6	0.0	6	0.0
154	beta-Caryophyllene epoxide	6	69.4	9	18.9	10	21.7	9	19.0	6	0.0	8	0.0	11	0.0	6	1.9	6	57.6	6	0.0
155	2-Phenylpropan-2-ol	7	49.8	6	0.0	9	40.8	10	56.4	6	1.9	9	0.0	7	0.0	7	8.9	8	25.3	6	5.1
156	1,1-Diethoxyhexane	6	62.8	7	20.9	8	39.4	8	27.8	6	0.0	6	36.2	8	0.0	7	0.0	7	0.0	8	0.0
157	2-Acetylpyridine	6	79.9	10	39.7	8	35.9	8	0.0	6	0.5	8	0.0	7	0.0	7	5.1	6	1.9	6	23.8
158	Cyclopentanone	6	0.0	7	28.8	9	31.2	6	33.7	6	0.0	7	17.9	8	0.0	7	12.8	6	40.6	8	21.4
159	2,3-Dimethylpyra-zine	7	72.7	8	8.6	9	36.8	9	0.0	6	0.0	9	0.0	8	0.0	6	6.4	6	55.4	6	5.0
160	Citronellyl isobutyr-ate	6	44.1	9	0.0	8	8.0	9	0.0	6	27.5	8	46.8	7	18.8	6	7.9	6	27.4	6	0.0
161	2-Acetylpyrrole	6	56.6		42.7	9	21.7	9	0.0	6	0.0	9	0.0	9	0.0	7	0.0	6	58.0	6	0.0
162	Diphenyl oxide	6	37.9	10	0.0	7	0.0	8	0.0	6	11.9	7	22.0	6	0.0	6	54.2	6	52.8	6	0.0
163	2-Butanol	6	50.7	9	0.0	9	32.4	10	38.8	7	0.0	9	15.5	6	10.1	6	0.0	8	30.9	6	0.0
164	alpha-picoline	6	65.0	10	12.3	7	36.3	8	48.8	6	0.0	8	0.0	7	0.0	6	10.8	6	0.0	6	0.0
165	1,8-Cineole	6	57.5	10	24.0	8	0.0	9	44.5	6	21.9	9	0.0	10	0.0	6	21.0	6	0.0	6	3.8
166	Thymol	11	62.5	13	0.0	11	27.2	9	0.0	8	0.0	9	18.6	12	0.0	9	7.3	8	55.9	9	0.0

167	Isoamyl 2-methyl-butyrate	7	32.9	6	26.9	6	0.0	8	0.0	6	19.7	8	61.0	6	0.0	6	0.0	6	30.5	7	0.0
168	Benzyl isovalerate	6	68.3	7	0.0	8	0.0	9	13.9	6	14.5	8	0.0	8	0.0	6	6.7	6	65.9	6	0.0
169	2,6-Dimethylpyra-zine	6	0.0	8	2.5	9	41.8	9	5.5	6	13.0	9	62.9	8	0.0	7	22.6	6	20.5	6	0.0
170	Cinnamic acid	8	32.2	9	27.2	11	5.3	14	15.4	7	5.7	13	44.9	11	0.0	6	3.4	8	32.9	7	0.0
171	Neryl acetate	6	46.2	12	13.9	8	17.9	7	22.0	6	13.0	6	0.0	7	0.0	6	11.4	6	30.6	6	10.3
172	Fenchyl acetate	6	45.4	10	20.8	10	16.2	9	44.6	6	0.0	9	0.0	7	21.5	6	14.3	6	0.0	8	1.9
173	Citronellyl tiglate	6	0.0	8	4.5	8	13.8	9	24.8	6	24.9	8	32.5	7	9.1	6	21.8	6	2.8	6	28.5
174	Linalyl acetate	6	78.1	9	5.9	9	24.6	7	0.0	6	0.0	7	0.0	7	0.0	6	6.9	6	32.8	6	13.7
175	Cyclohexanone	6	14.0	6	16.5	8	27.9	6	15.1	6	0.0	7	0.0	8	3.3	7	23.5	6	34.0	6	27.2
176	γ-Terpinene	7	48.5	9	16.2	7	12.6	7	7.0	6	0.0	6	5.1	7	19.8	7	2.2	6	50.0	6	0.0
177	Azelaic acid	8	31.7	9	0.0	10	16.8	11	38.8	7	0.0	10	35.6	8	5.5	6	10.0	7	22.3	7	0.0
178	1,1-Dimethoxyethane	6	34.2	7	22.2	9	25.2	10	9.6	6	0.0	9	54.3	8	14.4	6	0.0	8	0.0	6	0.0
179	Cuminal	6	0.0	9	0.0	11	0.0	11	0.0	6	9.0	10	64.8	6	0.0	6	0.0	6	70.0	6	14.2
180	2,5-Dimethylfuran	6	19.9	8	49.6	9	0.0	9	0.0	6	0.0	9	0.0	8	0.0	7	2.8	6	57.5	6	27.7
181	Dibenzyl disulfide	7	10.4	8	17.2	8	9.0	9	37.7	6	8.6	9	48.0	7	4.7	7	5.7	6	7.2	6	8.2
182	Butyl propionate	6	46.7	9	11.3	9	37.1	8	21.5	6	22.6	7	0.4	7	0.0	7	5.5	6	8.8	6	0.0
183	Tetradecanoic acid	7	4.2	7	28.5	6	13.4	6	28.4	6	16.4	7	43.6	9	13.4	7	0.0	6	0.0	6	5.0
184	Ethyl acetate	6	35.1	9	28.8	8	0.0	10	31.4	6	11.9	9	36.2	7	0.0	7	0.0	8	8.9	6	0.0
185	Citronellyl propio-nate	6	40.8	9	3.9	8	4.9	8	50.4	6	9.3	8	0.0	7	6.6	7	0.6	6	13.0	6	20.7
186	Phloretin	6	47.7	7	7.7	6	0.0	7	0.0	6	12.1	6	48.8	7	16.2	8	0.0	6	11.1	7	5.9
187	p-Cymene	6	41.7	6	0.0	9	0.0	6	54.0	6	0.0	7	24.9	6	0.0	6	0.0	6	28.3	7	0.0

188	(-)-Bornyl acetate	6	0.0	10	8.5	8	1.8	8	0.0	6	9.5	8	0.0	6	44.9	6	0.0	6	63.4	6	19.4
189	Cedryl acetate	7	73.4	8	3.0	9	14.8	8	0.0	6	5.6	8	0.0	8	0.0	6	0.0	6	45.8	6	0.0
190	Dibutyl disulfide	6	36.7	7	20.1	8	11.5	8	26.2	6	0.0	9	25.2	7	0.0	6	0.0	6	21.1	6	1.4
191	Dimethyl sulfide	7	64.4	9	22.1	9	0.0	10	0.0	8	0.0	9	0.0	8	0.0	7	0.0	7	52.4	7	0.0
192	2,6-Dimethylphenol	7	20.8	11	24.1	8	0.0	6	53.8	7	15.8	7	0.0	9	0.0	6	13.6	6	10.8	6	0.0
193	β-Caryophyllene	6	27.8	9	0.0	9	53.9	6	0.0	6	0.0	7	0.0	7	0.0	6	4.3	6	52.6	6	0.0
194	1.4-Dimethoxyben-zene	6	5.4	7	0.0	9	0.0	9	0.0	6	14.0	9	0.0	8	48.4	7	0.0	7	66.6	6	1.6
195	3,5-Dimethylphenol	6	0.0	6	7.5	9	7.3	6	76.8	6	0.0	7	0.0	6	27.7	7	15.6	6	0.0	7	0.0
196	Veratraldehyde	7	19.9	7	23.2	8	2.9	10	0.0	6	8.9	9	52.4	8	0.0	7	15.8	6	11.1	6	0.0
197	o-methoxycinnamal-dehyde	8	0.0	11	6.7	9	12.6	10	0.0	7	18.6	10	0.0	6	29.1	7	1.2	6	58.9	12	5.9
198	Dihydrocarvyl ace-tate	6	42.0	8	22.7	10	0.0	8	23.0	6	18.2	7	0.9	9	19.1	6	0.0	6	0.0	6	4.8
199	Cedrol	6	72.1	6	0.0	6	0.0	6	0.0	6	0.0	6	0.0	8	0.0	6	0.0	6	52.4	6	0.0
200	Anisic acid	7	14.1	9	16.2	8	0.0	11	69.6	6	23.9	11	0.0	11	0.0	6	0.0	6	0.0	6	0.0
201	Dodecane	6	0.0	8	0.0	8	1.9	8	41.0	6	0.0	8	26.3	7	39.6	6	7.2	6	2.3	6	3.5
202	2,5-Dimethylpyra-zine	7	65.1	7	0.0	9	34.6	9	11.6	6	0.0	9	0.0	8	0.0	6	1.1	6	0.0	6	9.1
203	Acetovanillone	6	3.8	8	20.7	8	0.0	8	0.0	6	13.6	8	21.7	10	0.0	6	0.0	7	23.4	10	37.9
204	2,5-Dimethylphenol	9	40.1	11	20.0	9	0.0	8	32.1	8	3.6	8	4.6	8	0.0	6	18.2	6	0.0	8	0.0
205	2,5-Dimethylfuran-3(2H)-one	6	12.6	9	4.5	9	5.0	7	30.0	6	6.0	8	41.6	7	0.0	8	0.0	6	0.0	6	17.8
206	Camphene	6	61.0	10	25.9	9	0.0	6	28.0	6	0.0	6	0.0	9	0.0	6	0.0	6	0.0	6	0.0
207	Decyl acetate	6	23.5	8	0.0	8	2.5	7	0.0	6	8.2	7	64.9	7	0.0	6	0.0	6	11.2	6	0.0
208	delta-hexalactone	6	0.0	10	0.0	10	0.0	10	20.8	6	0.0	10	27.6	8	0.0	7	14.7	6	31.3	6	15.6

209	Camphor	9	39.3	9	0.0	8	0.0	8	54.1	6	6.9	8	0.0	8	3.9	8	5.0	6	0.0	9	0.0
210	2-Butanone	6	14.9	9	0.0	9	0.0	9	7.7	6	0.0	9	16.4	7	0.0	6	17.8	6	22.4	6	29.6
211	Butane-1,3-diol	6	26.9	7	0.0	9	12.9	9	3.7	6	0.0	9	52.4	8	7.0	7	0.0	7	0.0	6	3.6
212	1-Butanol	6	0.0	7	0.0	8	0.4	9	40.8	6	0.0	8	56.8	7	0.0	7	0.0	7	0.0	6	0.0
213	Menthone	6	0.0	9	0.0	8	14.6	7	0.0	6	27.6	8	0.0	8	0.0	6	0.0	6	29.2	8	25.9
214	Terpinyl acetate	6	43.7	8	18.5	11	0.0	10	7.1	6	18.6	9	0.0	9	0.0	9	0.0	6	0.0	6	0.0
215	Ambrettolide	6	18.2	8	20.1	9	0.0	6	27.4	6	7.7	7	0.0	9	0.0	7	0.0	6	0.0	8	0.0
216	Acetone	9	18.4	11	0.0	12	3.4	12	0.0	11	0.0	12	1.2	11	0.0	9	24.7	9	12.4	8	0.0
217	Benzoic acid	6	14.6	9	0.0	8	23.1	11	3.2	6	0.0	9	0.0	7	0.0	7	0.0	7	0.0	6	12.7
218	Furaneol	6	0.0	7	19.2	8	0.0	8	0.0	6	7.6	8	3.2	7	0.0	7	9.5	6	0.0	6	0.0

Table S3: Minimal inhibitory concentration, with 90% relative growth inhibition against *P. palitans*, *P. hordei* and *A. westerdijkiae*.

	P. palitans	***P. hordei***	***A. westerdijkiae***
Acetaldehyde	128 µg/mL	128 µg/mL	64 µg/mL
E-cinnamaldehyde	128 µg/mL	64 µg/mL	64 µg/mL
Benzyl isothiocyanate	32 µg/mL	32 µg/mL	32 µg/mL
2,3-butanedione	64 µg/mL	64 µg/mL	64 µg/mL
Carvacrol	256 µg/mL	128 µg/mL	256 µg/mL
Hexanoic acid	512 µg/mL	256 µg/mL	256 µg/mL
Octanoic acid	256 µg/mL	128 µg/mL	128 µg/mL
Allyl isothiocyanate	16 µg/mL	32 µg/mL	16 µg/mL

Table S4: Synergistic potency, calculated according to the MuSyc principle, of two compounds against *P. palitans*, *P. hordei* and *A. westerdijkiae*. For each combination, the effect of drug 1 on drug 2 (α_{12}) and the effect of drug 2 on drug 1 (α_{21}) is calculated, with a value above 1 corresponding to synergistic potency and below 1 indicating antagonistic potency. In case of an indifferent result, α equals 1. Synergistic potency is calculated using the average relative growth of three repeats (*) or six repeats (°).

	P. palitans		***P. hordei***		***A. westerdijkiae***	
	α_{12}	α_{21}	α_{12}	α_{21}	α_{12}	α_{21}
Acetaldehyde + Allyl isothiocyanate*	1.0	1.0	1.0	1.0	1.0	1.0
Acetaldehyde + Benzyl isothiocyanate°	0.35	4.9	1.0	6.0	1.8	1.0
Acetaldehyde + 2,3-butanedione°	45.9	1.0	1.0	1.0	1.0	1.0
Acetaldehyde + Carvacrol°	1.0	1.0	38.4	1.0	1.0	435.9
Acetaldehyde + E-cinnamaldehyde*	1.0	1.0	1.0	1.0	1.0	1.0
Acetaldehyde + Hexanoic acid*	1.0	1.0	1.0	1.0	1.0	4.49
Acetaldehyde + Octanoic acid*	1.0	0.51	5.4	1.0	1.0	1.0
Benzyl isothiocyanate + 2,3-butanedione°	20.9	1.0	9.6	1.0	32.3	1.0
Benzyl isothiocyanate + Carvacrol°	131.4	1.0	15.2	2.7	1.0	1.0
Benzyl isothiocyanate + Hexanoic acid*	1.0	1.0	1.0	1.0	1.0	1.0
Benzyl isothiocyanate + Octanoic acid°	1.0	3.79	1.0	1.0	1.0	1.0
2,3-Butanedione + Allyl isothiocyanate°	0.0	1.0	1.0	1.0	1.0	0.0
2,3-butanedione + Carvacrol°	19.89	1.0	16.19	0.58	1.0	1.0

2,3-Butanedione + Hexanoic acid°	1.0	1.0	1.0	1.0	3.18	0.0
2,3-Butanedione + Octanoic acid°	1.0	1.0	1.0	1.0	1.0	1.0
Carvacrol + Allyl isothiocyanate°	1.0	1.0	1.0	1.0	1.0	1.0
Carvacrol + Hexanoic acid*	1.0	1.0	1.0	1.0	1.0	1.0
Carvacrol + Octanoic acid*	0.09	1.0	1.0	3.18	56.23	1.0
E-cinnamaldehyde + Allyl isothiocyanate°	1.0	1.0	1.0	1.0	1.0	1.0
E-cinnamaldehyde + Benzyl isothiocyanate*	1.0	1.0	753.01	4.73	1.0	1.0
E-cinnamaldehyde + Hexanoic acid*	1.0	1.0	1.0	1.0	6.25	1.0
E-cinnamaldehyde + Octanoic acid*	1.0	1.0	0.2	1.0	32.79	1.0
Hexanoic acid + Allyl isothiocyanate*	51.23	1.0	1.0	1.0	1.0	0.26
Hexanoic acid + Octanoic acid*	1.0	1.0	48.01	1.0	1.0	1.0
Octanoic acid + Allyl isothiocyanate*	1.0	1.0	0.41	1.0	1.0	0.25

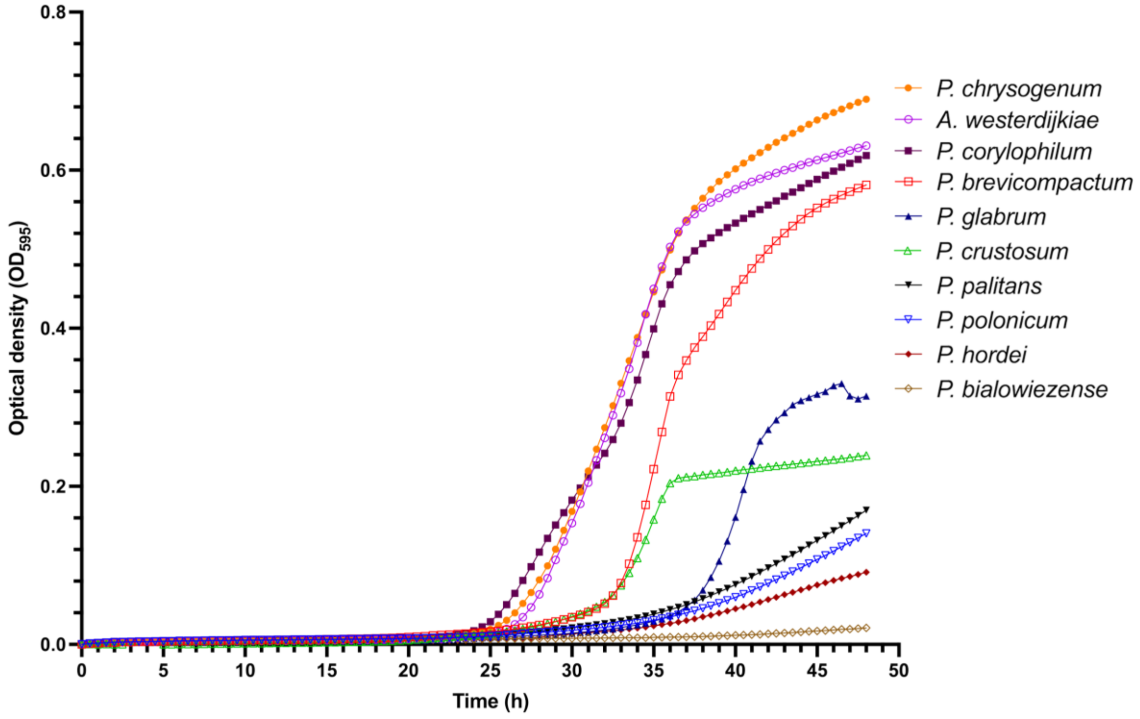

Figure S1: Growth curves of the ten moulds used in this research. The optical density was measured every 30 minutes using the Multiskan Microplate Spectrophotometer (Thermo Fisher). Each well of a 96-well plate contained 1000 spores, inoculated in semi solid YES medium, and six technical repeats were included for every strain. Temperature was kept at 26 °C and no shaking steps were included. The effect of the medium on the OD was taken into account by subtracting the measurement at 0h and the average of the six repeats is depicted in the graph.
